# Synthesis of a sucrose-based macrocycle with unsymmetrical monosaccharides "arms"

**DOI:** 10.3762/bjoc.14.50

**Published:** 2018-03-15

**Authors:** Karolina Tiara, Mykhaylo A Potopnyk, Sławomir Jarosz

**Affiliations:** 1 Institute of Organic Chemistry, Polish Academy of Sciences, Kasprzaka 44/52, 01-224 Warsaw, Poland

**Keywords:** chiral macrocycles, ring-closing metathesis, sucrose

## Abstract

An efficient methodology for the selective substitution of both terminal positions (C6 and C6’) in 1’,2,3,3’,4,4’-hexa-*O*-benzylsucrose with different unsaturated monosaccharide units is presented. Such a highly functionalized intermediate was cyclized under RCM conditions to afford a macrocyclic derivative containing a 31-membered ring in 26% yield.

## Introduction

Chiral macrocyclic compounds play an important role in supramolecular and biological systems [1–2]. Many of them serve as convenient receptors for cations [3], anions [4], ion pairs [5], neutral molecules [6] etc.

Binaphthols [7–9], amino acids [10], chiral diamines [11–12], carbohydrates [13], etc. are usually applied as building blocks for construction of such type of compounds.

We are engaged in the synthesis of such macrocyclic derivatives containing the most common natural disaccharide, sucrose [14–15]. Several different classes of macrocyclic derivatives, including: crown [16] and aza-crown [17–18] derivatives, macrocyclic dilactams [19–20], and ureas [21], were prepared in our laboratory.

Sucrose was also used by other groups as a precursor for the preparation of biodegradable polymers [22–24] and polymeric nanoparticles [25]. On the other hand, sucrose derivatives demonstrate antimicrobial and antitumor activities [26–27].

## Results and Discussion

**Scheme 1:**
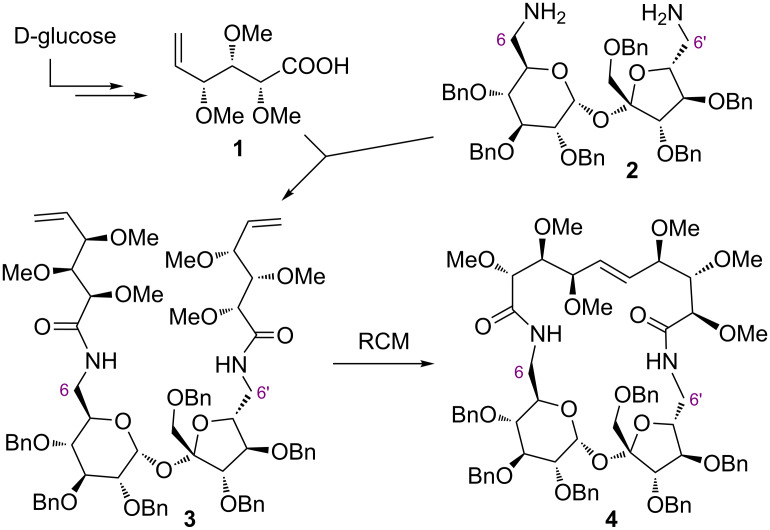
Synthesis of macrocyclic derivative **4**.

Recently, we have prepared sucrose-based macrocyclic derivative **4** in which the terminal positions of this disaccharide (C6 and C6’) are connected via a long polyhydroxylated bridge [28]. In this model study, both terminal positions in 6,6’-diamino-1’,2,3,3’,4,4’-hexa-*O*-benzyl-6,6’-dideoxysucrose (**2**) were elongated with the same polyhydroxylated unit **1** providing diamide **3**, which subsequently underwent cyclization under the chosen ring-closing metathesis (RCM) conditions [29–30] to give the 21-membered macrocycle **4** (Scheme 1).

**Figure 1:**
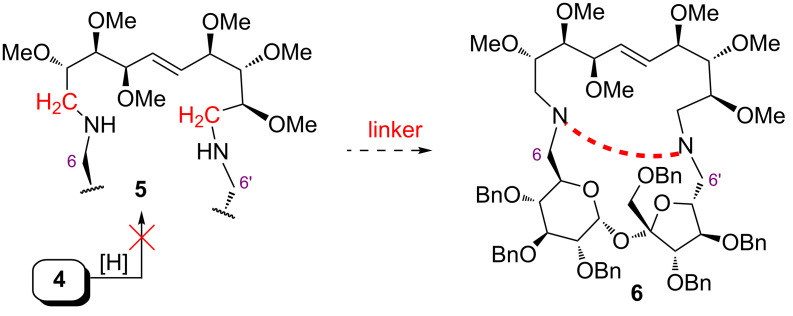
Possible route to sucrose cryptands **6**.

The reduction of the amide functions should lead to amines, which might be used as starting materials for the preparation of, e.g., cryptands **6** (Figure 1). All attempts, however, to reduce **4** to diamine **5** were unsuccessful.

**Figure 2:**
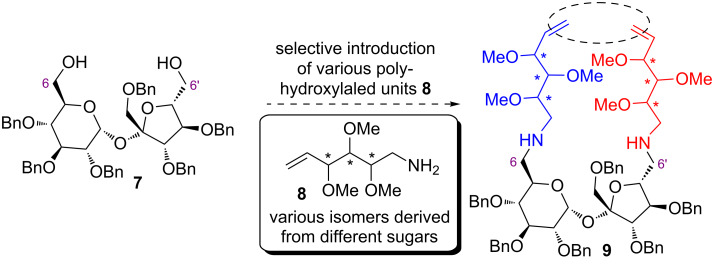
Possible route to dienes of type **9**.

We have decided, therefore, to elaborate another method leading to functionalized sucrose amines of type **9** (Figure 2) which will be obtained by a selective introduction of different fragments **8** (obtained from, e.g., glucose, mannose, etc.).

At this stage we were focused on the elaboration of a methodology allowing to introduce different fragments at the sucrose terminals. We chose, therefore, derivatives of methylated hexitols which are easy to prepare and are more convenient than benzyls in the interpretation of the NMR spectra.

**Scheme 2:**
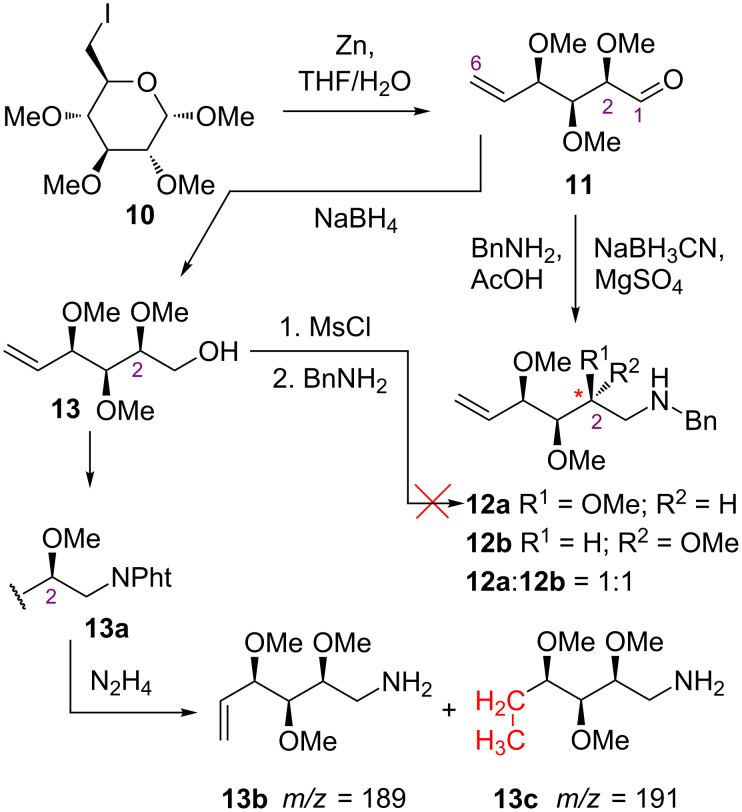
Unsuccessful attempts to amines **12a** and **13b**.

We faced, however, a serious problem in the synthesis of amines of type **8**. Treatment of aldehyde **11** – generated in situ from iodide **10** according to Vasellas' procedure [31–32] – with benzylamine under the reductive amination conditions afforded an inseparable mixture of two products differing in the configuration at the C2 center (**12a** and **12b**; Scheme 2); such a phenomenon – epimerization under these conditions – is known [33].

The alternative way to the desired amine **12a**, based on the S_N_2 reaction of the activated alcohol **13** [34–35] with benzylamine, also failed (Scheme 2).

The Mitsunobu approach to convert the hydroxy group into an amine function was also unsuccessful. Although **13** reacted with phthalimide gave the desired product **13a**, the deprotection of the amine function with hydrazine caused also reduction of the C5–C6 double bond; two peaks at 189 and 191 Da were observed in the MS spectrum of crude post-reaction mixture (for **13b** and **13c**, respectively).

**Scheme 3:**
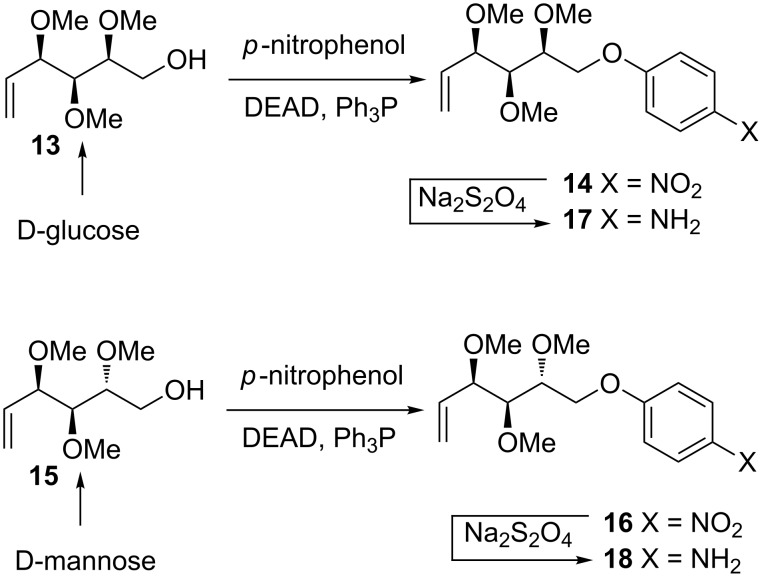
Syntheses of "elongated" amines **17** and **18**.

We reasoned, that the all these problems may be overcome by an elongation of alcohol **13** (derived from D-glucose) with a rigid fragment and we decided to introduce the phenyl ring. Treatment of alcohol **13** with *para*-nitrophenol under Mitsunobu conditions afforded the nitro compound **14** in 63% yield. Stereoisomeric alditol **15**, obtained from D-mannose, was converted analogously to **16** (in 60% yield). Both nitro compounds **14** and **16** were reduced to the corresponding amines **17** and **18** with sodium dithionite (Scheme 3).

The synthesis of the macrocycle with different arms at both terminal positions was initiated from 6’-*O*-*tert*-butyldiphenylsilyl-1’,2,3,3’,4,4’-hexa-*O*-benzylsucrose (**19**) readily available by a selective silylation of 1’,2,3,3’,4,4’-hexa-*O*-benzylsucrose (**7**) [36].

**Scheme 4:**
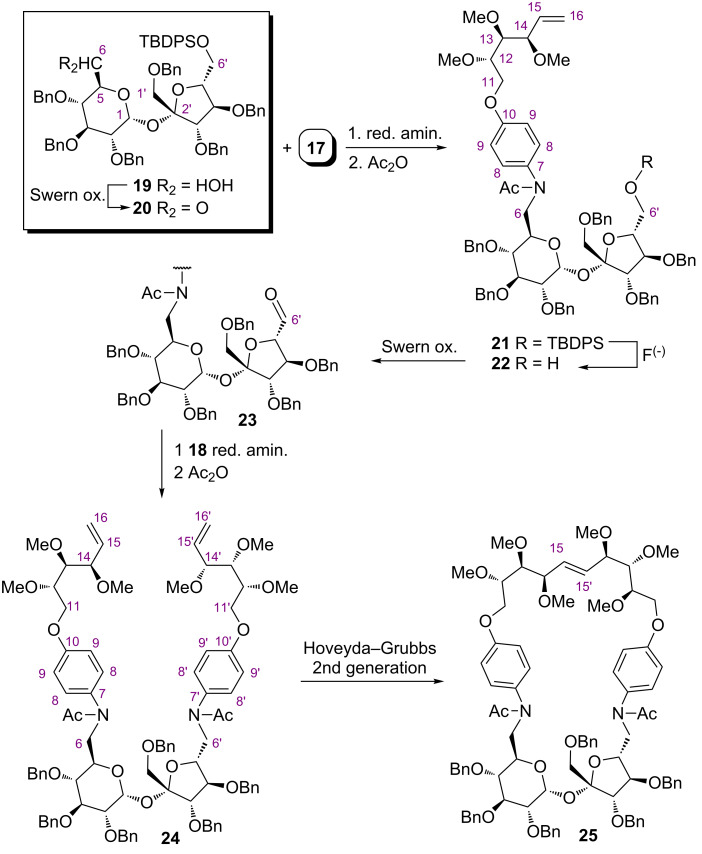
Synthesis of macrocycle **25**.

Aldehyde **20** [37] – obtained by Swern oxidation [38] of alcohol **19** – was reacted with amine **17** to afford the desired amine isolated as acetate **21** in 85% total yield. Removal of the TBDPS protecting group from the C6’-position gave alcohol **22** in 97% yield. Under the same "Swern oxidation–reductive amination–acetylation" conditions, alcohol **22** was converted into aldehyde **23**, which reacted further with amine **18**, furnishing diolefin **24** in 64% total yield. Cyclization of precursor **24** induced by the Hoveyda–Grubbs catalyst (II gen.) afforded the target macrocycle **25** in 26% yield (Scheme 4). The *E*-configuration of the newly created C=C-bond in the final product was proven by ^1^H NMR analysis (*J*_15-15’_ = 15.9 Hz).

## Conclusion

In summary, we proposed an efficient method of the synthesis of a 31-membered macrocycle with sucrose scaffold. The proposed methodology allows for the regioselective introduction of various polyhydroxylated unsaturated fragments (derived from different sugars) at either terminal position of sucrose which undergo an efficient cyclization under the RCM conditions. Although, for practical reasons, the method was elaborated for the methylated derivatives of hextitol pendants it might be, eventually, applied also for synthons with other protecting groups.

## Experimental

### General

The NMR spectra were recorded with a Varian VNMRS 600 MHz spectrometer for solutions in CDCl_3_ at 25 °C. The ^13^C NMR data for compound **25** were recorded with a Varian VNMRS 500 MHz spectrometer. The structures were assigned, whenever necessary, with the help of 2D correlation experiments (COSY, HSQC, HMBC). Chemical shifts were reported with reference to TMS. Optical rotations were measured with a Jasco P 1020 polarimeter (sodium light) in chloroform at room temperature. Mass spectra were recorded with a Synapt G2-S HDMS (Waters Inc) mass spectrometer equipped with an electrospray ion source and a q-TOF type mass analyzer. The instrument was controlled and recorded data were processed using the MassLynx V4.1 software package (Waters Inc). Thin-layer chromatography (TLC) was performed on silica gel plates coated with fluorescent indicator. Column chromatography was performed on silica gel (Merck, 230–400 mesh). Organic solutions were dried over anhydrous MgSO_4_.

### Procedure for the synthesis of nitro compounds **14** and **16**

To a solution of alcohol **13** (310 mg, 1.63 mmol) in dry THF (12 mL) and toluene (4 mL), Ph_3_P (1.28 g, 4.89 mmol) and *para*-nitrophenol (340 mg, 2.44 mmol) were added. After stirring during 15 min, DEAD (384 μL, 2.44 mmol) was added dropwise. The reaction mixture was stirred for 3 h at room temperature and then partitioned between diethyl ether (20 mL) and 5% NaOH aqueous solution (30 mL). The layers were separated and the aqueous one extracted with diethyl ether (3 × 15 mL). The combined organic solutions were washed with water (15 mL) and brine (15 mL), dried, concentrated, and the resulting residue was purified by flash chromatography (hexanes–ethyl acetate, 70:30) to afford pure product **15** (318 mg, 1.02 mmol, 63%) as a white solid. TLC [hexanes–AcOEt (2:1)]: *R*_f_ = 0.3; [α]_D_^22^ +28.5; ^1^H NMR δ 8.18 (d, *J* = 9.3 Hz, 2H, ArH), 6.99 (d, *J* = 9.3 Hz, 2H, ArH), 6.03 (ddd, *J*_5,4_ = 6.0 Hz, *J*_5,6_ = 10.7 Hz, *J*_5.6_ = 17.3 Hz, 1H, H-5), 5.39 (m, 2H, H-6, H-6’), 4.95 (dd, *J*_4,5_ = 6.2 Hz, *J*_4,3_ = 7.3 Hz, 1H, H-4), 3.63–3.54 (m, 3H, 2×H-1, H-2), 3.53 (dd, *J*_3,2_ = 2.5 Hz, 1H, H-3), 3.49 (s, 3H, OMe), 3.42 (s, 3H, OMe), 3.29 (s, 3H, OMe) ppm; ^13^C NMR δ 162.73 (C-Ar), 141.57 (C-Ar), 133.98 (C-5), 125.82 (2 × C-Ar), 119.07 (C-6), 115.51 (2 × C-Ar), 82.30 (C-3), 78.61 (C-2), 77.54 (C-4), 70.74 (C-1), 61.20 (OMe), 59.09 (OMe), 59.08 (OMe) ppm; HRMS (ESI) [M + Na]^+^: calcd for C_15_H_21_NO_6_Na, 334.1257; found, 334.1256; anal. calcd for C_15_H_21_NO_6_ (311.33): C, 57.87; H, 6.80; N, 4.50; found: C, 57.65; H, 6.79; N, 4.57.

**Nitro compound 16** was obtained as a white solid in 60% yield (295 mg, 0.95 mmol), using the same procedure, from alcohol **15** (300 mg, 1.58 mmol), Ph_3_P (1.24 g, 4.73 mmol), *para*-nitrophenol (329 mg, 2.37 mmol), and DEAD (371 μL, 2.37 mmol). TLC [hexanes–AcOEt (2:1)]: *R*_f_ = 0.3. [α]_D_^22^ −18.9; ^1^H NMR δ 8.21 (d, *J* = 10 Hz, 2H, ArH), 7.03 (d, *J* = 10 Hz, 2H, ArH), 5.92 (ddd, *J*_5,4_ = 7.8 Hz, *J*_5,6_ = 10.4 Hz, *J*_5.6’_ = 17.3 Hz, 1H, H-5), 5.41–5.34 (m, 2H, 2 × H-6), 4.39 (dd, *J*_1,1_ = 10.3 Hz, *J*_1,2_ = 2.3 Hz, 1H, H-1), 4.19 (dd, *J*_1,1_ = 10.3 Hz, *J*_1,2_ = 4.7 Hz, 1H, H-1), 3.86 (m, 1H, H-4), 3.75 (m, 1H, H-2), 3.49 (s, 3H, OMe), 3.43 (m, 4H, OMe, H-3), 3.34 (s, 3H, OMe) ppm; ^13^C NMR δ 163.82 (C-Ar), 141.61 (C-Ar), 135.54 (C-5), 125.89 (2 × C-Ar), 118.71 (C-6), 114.58 (2 × C-Ar), 82.59 (C-3), 81.81 (C-4), 78.81 (C-2), 67.56 (C-1), 61.25, 58.45, 56.72 (3 × OMe) ppm; HRMS (ESI) [M + Na]^+^: calcd for C_15_H_21_NO_6_Na 334.1257; found: 334.1256; anal. calcd for C_15_H_21_NO_6_ (311.33): C, 57.87; H, 6.80; N, 4.50; found: C, 57.69; H, 6.81; N, 4.50.

### Synthesis of amino compounds **17** and **18**

To a solution of nitro compound **14** or **16** (232 mg, 0.74 mmol) in aq ethanol (14 mL, 1:1 v/v), K_2_CO_3_ (304 mg, 2.22 mmol) and Na_2_S_2_O_4_ (322 mg, 1.85 mmol) were added, and the mixture was stirred for 30 min at rt. Ethyl acetate (15 mL) was added, the layers were separated, and the aqueous one extracted with ethyl acetate (3 × 8 mL). The combined organic solutions were dried, concentrated, and the crude product **17** or **18** was used in the next step without further purification.

### Synthesis of compound **21**

A solution of amine **17** (203 mg, 0.72 mmol) in DCM (10 mL) was added to a solution of aldehyde **20** (202 mg, 0.18 mmol; prepared from alcohol **19** as described in our previous paper [37]) in DCM (10 mL) containing acetic acid (41 μL, 0.72 mmol) and MgSO_4_ (≈200 mg), and the mixture was stirred for 1 h at rt. Then, NaBH_3_CN (17 mg, 0.72 mmol) was added and stirring was continued overnight. Water (20 mL), 0.1 M solution of NH_3_ (2 mL), and DCM (15 mL) were added, the layers were separated, and the aqueous one extracted with DCM (3 × 10 mL). The combined organic solutions were washed with water (10 mL) and brine (10 mL), dried and concentrated. The residue was dissolved in 1,4-dioxane (10 mL) to which DMAP (4 mg, 0.04 mmol), Et_3_N (126 μL, 0.90 mmol), and acetic anhydride (51 μL, 0.54 mmol) were added and the mixture was stirred overnight at 100 °C. After cooling to rt, water (15 mL) and DCM (15 mL) were added, the layers were separated, and the aqueous one was extracted with DCM (3 × 10 mL). Combined organic solutions were washed with water (10 mL) and brine (10 mL), dried, concentrated, and the crude productwas purified by flash chromatography (hexanes–ethyl acetate, 80:20 to 50:50) to afford **21** (219 mg, 0.15 mmol, 85%) as a colorless oil. TLC [hexanes–AcOEt (2:1)]: *R*_f_ = 0.2; [α]_D_^22^ +13.6; ^1^H NMR δ 7.66 (m, 4H, ArH), 7.33–7.15 (m, 36H, ArH), 7.05 (d, *J*_9,8_ = 8.7 Hz, 2H, 2 × H-9), 6.77 (d, *J*_8,9_ = 8.7 Hz, 2H, 2 × H-8), 5.92 (ddd, *J*_15,14_ = 6.0 Hz, *J*_15,16_ = 10.6 Hz, *J*_15,16_ = 17.2 Hz, 1H, H-15), 5.76 (d, *J*_1,2_ = 3.3 Hz, 1H, H-1), 5.30 (d, *J*_16,15_ = 17.2 Hz, 1H, H-16), 5.21 (d, *J*_16,15_ = 10.6 Hz, 1H, H-16), 4.79 (d, *J* = 11.1 Hz, 1H, benzylic H), 4.77–4.71 (m, H-14, 3H, 2 × benzylic H), 4.63 (d, *J* = 11.8 Hz, 1H, benzylic H), 4.62 (d, *J* = 11.4 Hz, 1H, benzylic H), 4.56 (d, *J* = 11.9 Hz, 1H, benzylic H), 4.50 (d, *J* = 11.8 Hz, 1H, benzylic H), 4.46 (d, *J* = 10.8 Hz, 1H, benzylic H), 4.43 (d, *J* = 11.8 Hz, 1H, benzylic H), 4.42–4.37 (m, H-3’, 4H, 3 × benzylic H), 4.23 (dd, *J*_4’,5’_ = 5.9 Hz, *J*_4’,3’_ = 6.2 Hz, 1H, H-4’), 4.19 (m, 1H, H-5), 4.07 (dd, *J*_5’,6’_ = 11.7 Hz, *J*_5’,4’_ = 5.9 Hz, 1H, H-5’), 3.96 (m, 3H, 2 × H-6’, H-6), 3.83 (dd, *J*_1’,1’_ = 10.3 Hz, 1H, H-1’), 3.81 (m, 1H, H-3), 3.66 (dd, *J*_5,6_ = 6.7 Hz, *J*_6,6_ = 14.0 Hz, 1H, H-6), 3.61–3.51 (m, 4H, 2 × H-11, H-12, H-1’), 3.45 (s, 3H, OMe), 3.43 (dd, *J* = 2.25 Hz, *J* = 7.2 Hz, 1H, H-13), 3.39 (s, 3H, OMe), 3.38 (m, 1H, H-2), 3.22 (s, 3H, OMe), 3.19 (m, 1H, H-4), 1.66 (s, 3H, OAc), 1.05 (s, 9H, *t-*Bu) ppm; ^13^C NMR δ 170.84 (C=O), 156.83 (C-10), 138.73, 138.68, 138.42, 138.36, 138.24, 137.88 (6C_quat_, 6 × Ph), 137.49 (C-7), 135.57 (2C-Ph), 135.51 (2C-Ph), 134.97 (C-15), 133.44, 133.28 (2C_quat_, 2 × Ph), 129.63 (2C-Ph), 129.58 (2C-Ph), 129.24 (2 × C-9), 128.36–127.29 (m, 32C-Ar), 118.42 (C-16), 116.32 (2 × C-8), 105.17 (C-2’), 90.18 (C-1), 83.94 (C-3’), 83.59 (C-4’), 82.57 (C-13), 81.97 (C-5’), 81.47 (C-3), 80.01 (C-2), 79.85 (C-4), 78.66 (C-12), 77.21 (C-14), 75.41, 73.90, 73.41, 72.98, 72.35, 71.98 (6 × OBn), 71.02 (C-11), 70.37 (C-1’), 69.51 (C-5), 65.46 (C-6’), 61.10, 59.05, 59.05 (3 × OMe), 50.50 (C-6), 26.93 [3C, SiC(*C*H_3_)_3_], 22.90 (*C*H_3_CO_2_), 19.27 (C_quat_-*t-*Bu) ppm; HRMS (ESI) [M + Na]^+^ calcd for C_87_H_99_O_15_SiNa, 1448.6669; found, 1448.6682; anal. calcd for C_87_H_99_NO_15_Si (1426.83): C, 73.24; H, 6.99; N, 0.98; found: C, 73.23; H, 7.10; N, 1.08.

### Synthesis of alcohol **22**

A solution of TBAF (113 mg, 0.43 mmol) in THF (5 mL) was added to a solution of compound **21** (205 mg, 0.14 mmol) in dry THF (10 mL), and the resulting mixture was stirred for 1 h at rt, and concentrated. The crude product was purified by flash chromatography (hexanes–ethyl acetate, 50:50 to 40:60) to afford **22** (166 mg, 0.14 mmol, 97%) as a colorless oil. TLC [hexanes–AcOEt (1:2)]: *R*_f_ = 0.3; [α]_D_^22^ +27.3; ^1^H NMR δ 7.32–7.21 (m, 30H, ArH), 7.10 (d, *J_9_*_,8_ = 8.7 Hz, 2H, 2 × H-9), 6.84 (d, *J*_8,9_ = 8.7 Hz, 2H, 2 × H-8), 5.98 (ddd, *J*_15,14_ = 6.0 Hz, *J*_15,16_ = 10.4 Hz, *J*_15,16_ = 17.3 Hz, 1H, H-15), 5.52 (d, *J*_1,2_ = 3.0 Hz, 1H, H-1), 5.36 (d, *J*_16,15_ = 17.3 Hz, 1H, H-16), 5.28 (d, *J*_16,15_ = 10.4 Hz, 1H, H-16), 4.81 (m, H-14, 3H, 2 × benzylic H), 4.70 (d, *J* = 11.6 Hz, 1H, benzylic H), 4.66–4.57 (m, 4H, 4 × benzylic H), 4.52–4.48 (m, 3H, 3 × benzylic H), 4.42–4.38 (m, 2H, H-3’, benzylic H), 4.35 (d, *J* = 11.9 Hz, 1H, benzylic H), 4.32–4.28 (m, 2H, H-5, H-4’), 4.02–3.95 (m, 3H, H-3, H-5’, H-6), 3.85 (dd, *J*_6,6_ = 14.1 Hz, *J*_6,5_ = 5.8 Hz, 1H, H-6), 3.77–3.72 (m, 2H, H-1’,H-6’), 3.68–3.48 (m, 5H, 2 × H11, H-12, H-1’, H-6’), 3.45 (m, 5H, H-2, H-13, OMe), 3.40 (s, 3H, OMe), 3.37 (br s, 1H, OH), 3.26 (m, 1H, H-4), 3.25 (s, 3H, OMe), 1.78 (s, 3H, OAc) ppm; ^13^C NMR δ 171.83 (C=O), 156.89 (C-10), 138.54, 138.48, 138.39, 138.16, 138.09, 137.72 (C_quat_, 6 × Ph), 137.46 (C-7), 135.01 (C-15), 129.18 (2 ×C-9), 127.40–128.37 (m, 30C-Ar), 118.47 (C-16), 116.31 (2 × C-8), 104.61 (C-2’), 90.64 (C-1), 83.71 (C-3’), 82.57 (C-13), 82.01 (C-5’), 81.46 (C-3), 81.23 (C-4’), 79.71 (C-2), 79.50 (C-4), 78.68 (C-12), 77.26 (C-14), 75.43, 74.10, 73.36, 72.78, 72.67, 72.53 (6 × OBn), 71.04 (C-11), 70.76 (C-1’), 70.15 (C-5), 62.23 (C-6’), 61.13, 59.07, 59.07 (3 × OMe), 50.15 (C-6), 22.93 (*C*H_3_CO_2_) ppm; HRMS (ESI) [M + Na]^+^ calcd for C_71_H_81_NO_15_Na, 1210.5504; found, 1210.5544; anal. calcd for C_71_H_81_NO_15_ (1187.42): C, 71.76; H, 6.87; N, 1.18; found: C, 71.93; H, 7.01; N, 1.08.

### Synthesis of diolefin **24**

To a cooled solution (−78 °C) of oxalyl chloride (39 μL, 0.46 mmol) in DCM (10 mL), a solution of DMSO (93 μL, 1.30 mmol) in DCM (3 mL) was added within 5 min. After 10 min alcohol **22** (155 mg, 0.13 mmol) in DCM (3 mL) was added dropwise and stirring was continued for 10 min at −78 °C. Then, Et_3_N (145 μL, 1.04 mmol) was added and the mixture was allowed to attain rt. Water (7 mL) was added, the organic layer was separated, dried, and concentrated. Crude aldehyde **23** was dissolved in DCM (10 mL) and this solution was added to a solution of amine **18** (147 mg, 0.52 mmol), acetic acid (30 μL, 0.52 mmol), and MgSO_4_ (≈200 mg) in DCM (10 mL), and the resulting mixture was stirred for 1 h at rt. Then, NaBH_3_CN (12 mg, 0.20 mmol) was added and the stirring was continued overnight. Water (20 mL), 0.1 M solution of NH_3_ (2 mL), and DCM (15 mL) were added, the layers were separated, and the aqueous one extracted with DCM (3 × 10 mL). Combined organic solutions were washed with water (10 mL) and brine (10 mL), dried, concentrated, and the residue was dissolved in 1,4-dioxane (10 mL). DMAP (3 mg, 0.03 mmol), Et_3_N (91 μL, 0.65 mmol), acetic anhydride (37 μL, 0.39 mmol) were added, and the mixture was stirred overnight at 100 °C. After cooling, water (15 mL) and DCM (15 mL) were added, the layers were separated, and the aqueous one was extracted with DCM (3 × 10 mL). Combined organic solutions were washed with water (10 mL) and brine (10 mL), dried, concentrated, and the product was isolated by flash chromatography (hexanes–ethyl acetate, 25:75) to afford **24** (120 mg, 0.08 mmol, 64%) as a colorless oil. TLC [hexanes–AcOEt (1:3)]: *R*_f_ = 0.25; [α]_D_^22^ +32.7; ^1^H NMR δ 7.25–7.06 (m, 30H, ArH), 7.06–6.95 (m, 4H, 2 × H-9, 2 × H-9’), 6.79–6.72 (m, 4H, 2 × H-8, 2 × H-8’), 5.91 (m, 1H, H-15), 5.82 (m, 1H, H-15’), 5.41 (d, *J*_1,2_ = 3.2 Hz, 1H, H-1), 5.31–5.16 (m, 4H, 2 × H-16, 2 × H-16’), 4.78 (*J* = 11.1 Hz, 1H, benzylic H), 4.75–4.69 (m, 2H, benzylic H, H-14), 4.57–4.45 (m, 4H, 4 × benzylic H), 4.43–4.36 (m, 3H, 2 × benzylic H, H-12’), 4.35–4.26 (m, 3H, 2 × benzylic H, H-4’), 4.18 (m, 1H, H-5), 4.12 (dd, *J*_11’,11’_ = 10.0 Hz, *J*_11’,12’_ = 2.1 Hz, 1H, H-11’), 4.06–4.02 (m, 2H, H-5’, H-3’), 3.94–3.82 (m, 4H, 2 × H-6, H-6’, H-11’), 3.79–3.70 (m, 3H, H-6’, H-14’, H-11,), 3.63–3.56 (3H, m, H-3, H-11, H-12), 3.54–3.43 (m, 4H, 2 × H-1’, H-13, H-13’), 3.40 (s, 3H, OMe), 3.37 (s, 3H, OMe), 3.33–3.28 (m, 7H, 2×OMe, H-2), 3.25 (s, 3H, OMe), 3.21 (m, 1H, H-4), 3.16 (s, 3H, OMe), 1.67 (s, 3H, OAc), 1.64 (s, 3H, OAc) ppm; ^13^C NMR δ 171.13, 170.77 (2 × C=O), 157.98, 156.79 (C-10, C-10’), 138.97, 138.79, 138.46, 138.32, 138.32, 137.99, 137.89, 136.04 (Cquat, 6 × Ph, C-7, C-7’), 135.74 (C-15’), 135.09 (C-15), 129.52, 129.44 (2 × C-9, 2 × C-9’), 128.31–127.25 (m, 30 × C-Ar), 118.48, 118.43 (C-16, C-16’), 116.23, 115.18 (2 × C-8, 2 × C-8’), 104.93 (C-2’), 89.27 (C-1), 84.05 (C-3’), 83.54 (C-4’), 82.56 (C-13), 81.81 (C-14’), 81.73 (C-12’), 80.13 (C-2), 79.75 (C-13’), 79.23 (C-4), 78.81 (C-3), 78.73 (C-12), 77.54 (C-14), 77.48 (C-5’), 75.22, 73.95, 73.26, 72.67, 72.27, 72.25 (6 × OBn), 71.16 (C-11), 71.04 (C-1’), 70.13 (C-5), 66.55 (C-11’), 61.20, 61.10, 59.05, 59.01, 58.24, 56.70 (6 × OMe), 52.08 (C-6’), 49.66 (C-6), 22.90, 22.75 (2 × *C*H_3_CO_2_) ppm; HRMS (ESI) [M + Na]^+^calcd for C_88_H_104_N_2_O_19_Na, 1515.7153; found, 1515.7131; anal. calcd for C_88_H_104_N_2_O_19_ (1493.80): C, 70.76; H, 7.02; N, 1.88; found: C, 70.62; H, 7.10; N, 1.90.

### Synthesis of macrocyclic compound **25**

To a solution of diene **24** (85.0 mg, 0.060 mmol) in degassed, anhydrous toluene (10 mL), Hoveyda–Grubbs catalyst 2nd generation (3.7 mg, 0.006 mmol) was added, and the mixture was stirred and heated at 95 °C for 48 h. The mixture was concentrated and the product was purified by flash chromatography (hexanes–ethyl acetate, 15:85) to give macrocycle **25** (22.8 mg, 0.016 mmol, 26%) as a white amorphous foam. TLC [hexanes–AcOEt (1:5)]: *R*_f_ = 0.2; [α]_D_^22^ +13.2; ^1^H NMR δ 7.40–7.02 (m, 36H, ArH), 6.60 (m, 2H, ArH), 5.97 (dd, *J*_15’,15_ = 15.9 Hz, *J*_15’,14’_ = 6.4 Hz, 1H, H-15’), 5.74–5.68 (m, 2H, H-1, H-15), 4.87 (m, 1H, H-14’), 4.70 (d, *J* = 12.0 Hz, 1H, benzylic H), 4.66 (m, 1H, H-5’), 4.59 (d, *J* =10.8 Hz, 1H, benzylic H), 4.58 (d, *J* =11.7 Hz, 1H, benzylic H), 4.54 (d, *J* = 10.8 Hz, 1H, benzylic H), 4.50 (d, *J* = 11.2 Hz, 1H, benzylic H), 4.48 (d, *J* = 11.7 Hz, 1H, benzylic H), 4.48 (d, *J* = 11.7 Hz, 1H, benzylic H), 4.46 (dd, *J* = 7.3 Hz, *J* = 6.0 Hz, 1H, H-4’), 4.41–4.33 (m, 4H, 3 × benzylic H, H-6’), 4.30 (d, *J* = 11.0 Hz, 1H, benzylic H), 4.15 (d, *J* = 10.8 Hz, 1H, benzylic H), 4.02–3.96 (m, 2H, H-6, H-3’), 3.95–3.90 (m, 2H, H-1’, H-14), 3.82 (m, 1H, H-5), 3.77 (m, 1H, H-11’), 3.72–3.61 (5H, H-12, H-12’, H-1’, H-11, H-11’), 3.58 (m, 1H, H-11), 3.55–3.51 (m, 2H, H-13, H-13’), 3.50 (s, 3H, OMe), 3.49 (s, 3H, OMe), 3.41 (s, 3H, OMe), 3.41 (s, 3H, OMe), 3.39 (s, 3H, OMe), 3.37 (s, 3H, OMe), 3.35 (m, 1H, H-6’), 3.23 (dd, *J*_2,3_ = 6.7 Hz, *J*_2,1_ = 4.0 Hz, 1H, H-2), 3.14 (dd, *J*_3,4_ = 9.5 Hz, 1H, H-3), 3.07 (m, 1H, H-6), 3.00 (dd, *J*_4,5_ = 9.0 Hz, 1H, H-4), 1.90 (s, 3H, OAc), 1.73 (s, 3H, OAc) ppm; ^13^C NMR (125 MHz) δ 171.21, 170.78 (2 × C=O), 157.56, 157.49 (C-10, C-10’), 138.50, 138.50, 138.39, 138.13, 138.00, 137.83, 137.53, 137.36 (C_quat_, 6 × Ph, C-7, C-7’), 131.73 (C-15’), 130.50 (C-15), 129.46, 129.02 (2 × C-9, 2 × C-9’), 128.98–128.41 (m, 30 × C-Ar), 119.31, 115.03 (2 × C-8, 2 × C-8’), 105.77 (C-2’), 91.08 (C-1), 84.23 (C-3’), 84.23 (C-4’), 83.73 (C-13), 82.10 (C-13’), 81.79 (C-3), 81.27 (C-14), 79.68 (C-12’), 79.41 (C-5’), 79.41 (C-2), 78.95 (C-14’), 78.32 (C-12), 76.75 (C-4), 75.65, 73.72, 73.43, 73.28, 72.39, 72.23 (6 × OBn), 70.69 (C-11), 70.56 (C-5), 69.05 (C-1’), 67.27 (C-11’), 61.22, 59.54, 59.12, 59.01, 58.66, 57.07 (6 × OMe), 56.43 (C-6’), 49.57 (C-6), 23.06, 22.68 (2 × *C*H_3_CO_2_) ppm; HRMS (ESI) [M + Na]^+^ calcd for C_86_H_100_N_2_O_19_Na, 1487.6857; found, 1487.6971; anal. calcd for C_86_H_100_N_2_O_19_ (1465.74): C, 70.47; H, 6.88; N, 1.91; found: C, 70.62; H, 6.96; N, 1.84.

## Supporting Information

File 1 Copies of NMR spectra.
