# Supplementary material for: Synthesis of a sucrose-based macrocycle with unsymmetrical monosaccharides "arms"
Source: Beilstein J Org Chem. 2018 Mar 15;14:634–41. doi: 10.3762/bjoc.14.50 (PMC5870162; doi:10.3762/bjoc.14.50)
Supplement: File 1 — Copies of NMR spectra. [file Beilstein_J_Org_Chem-14-634-s001.pdf]

Supporting Information

for

Synthesis of a sucrose-based macrocycle with unsymmetrical monosaccharides "arms"

Karolina Tiara, Mykhaylo A. Potopnyk and Sławomir Jarosz*

Address: Institute of Organic Chemistry, Polish Academy of Sciences, Kasprzaka 44/52, 01-224 Warsaw, Poland

Email: Sławomir Jarosz - slawomir.jarosz@icho.edu.pl

*Corresponding author

Copies of NMR spectra

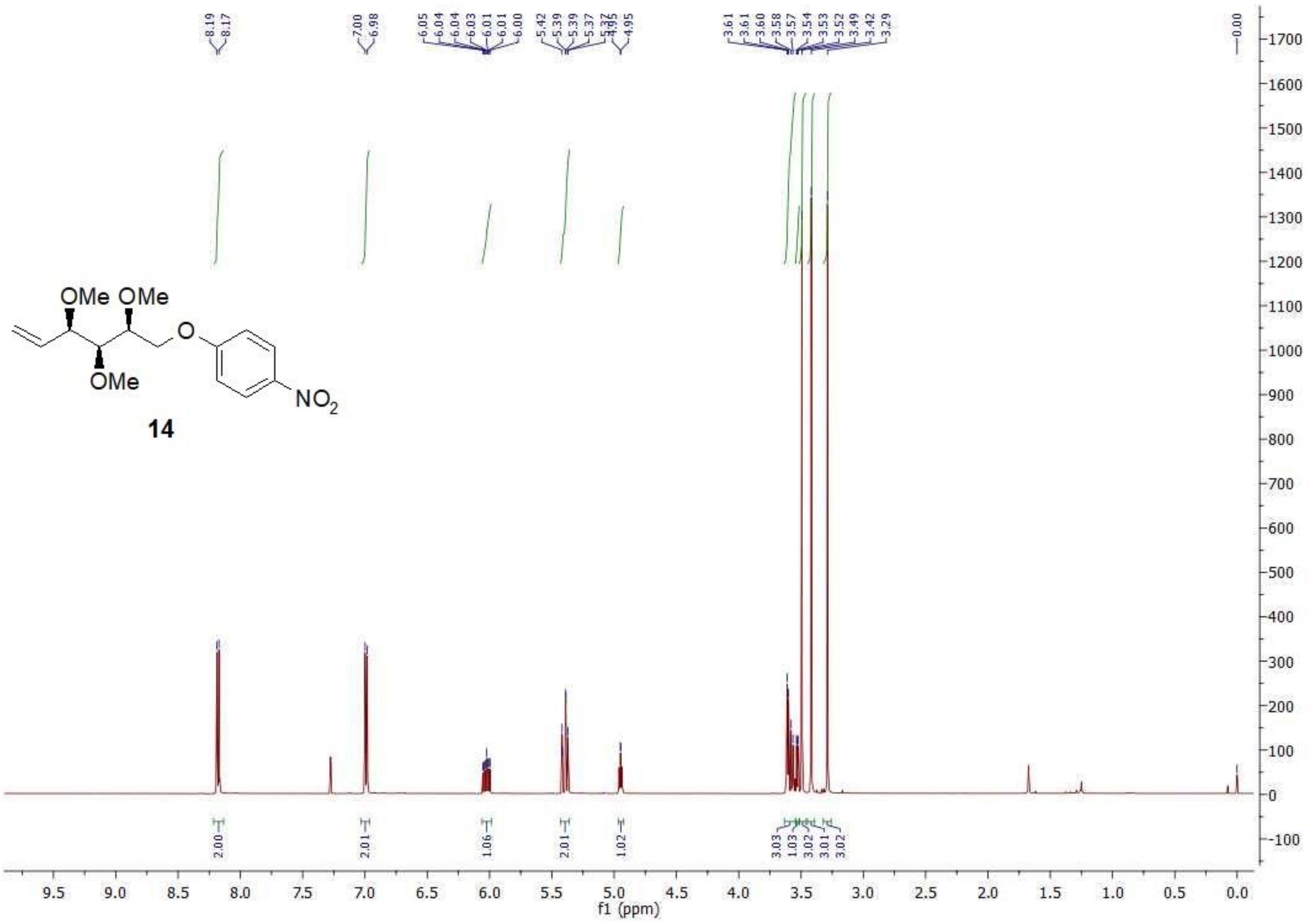

Figure S1 ^{1}H NMR spectrum of compound **14**.

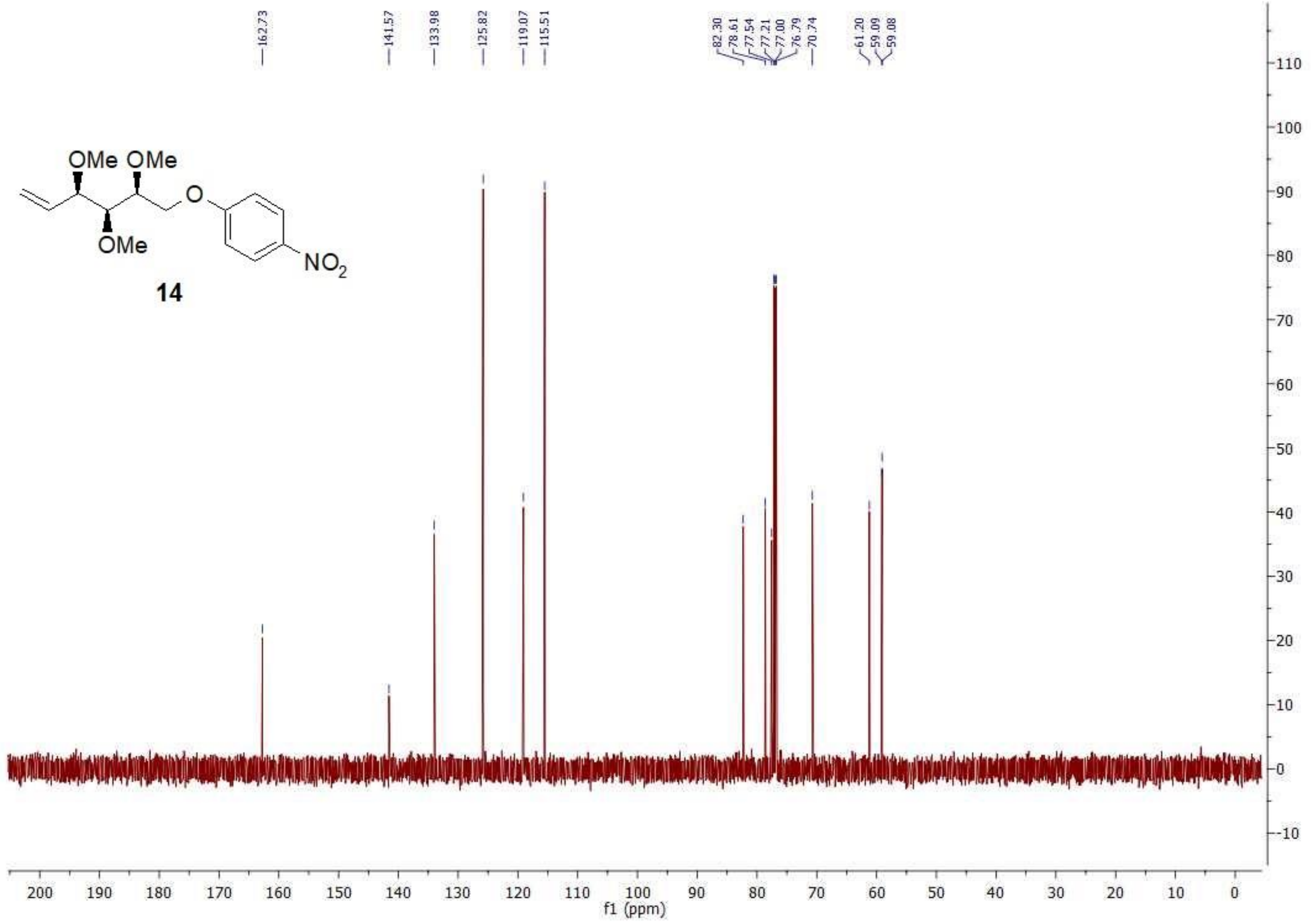

Figure S2 ^{13}C NMR spectrum of compound **14**.

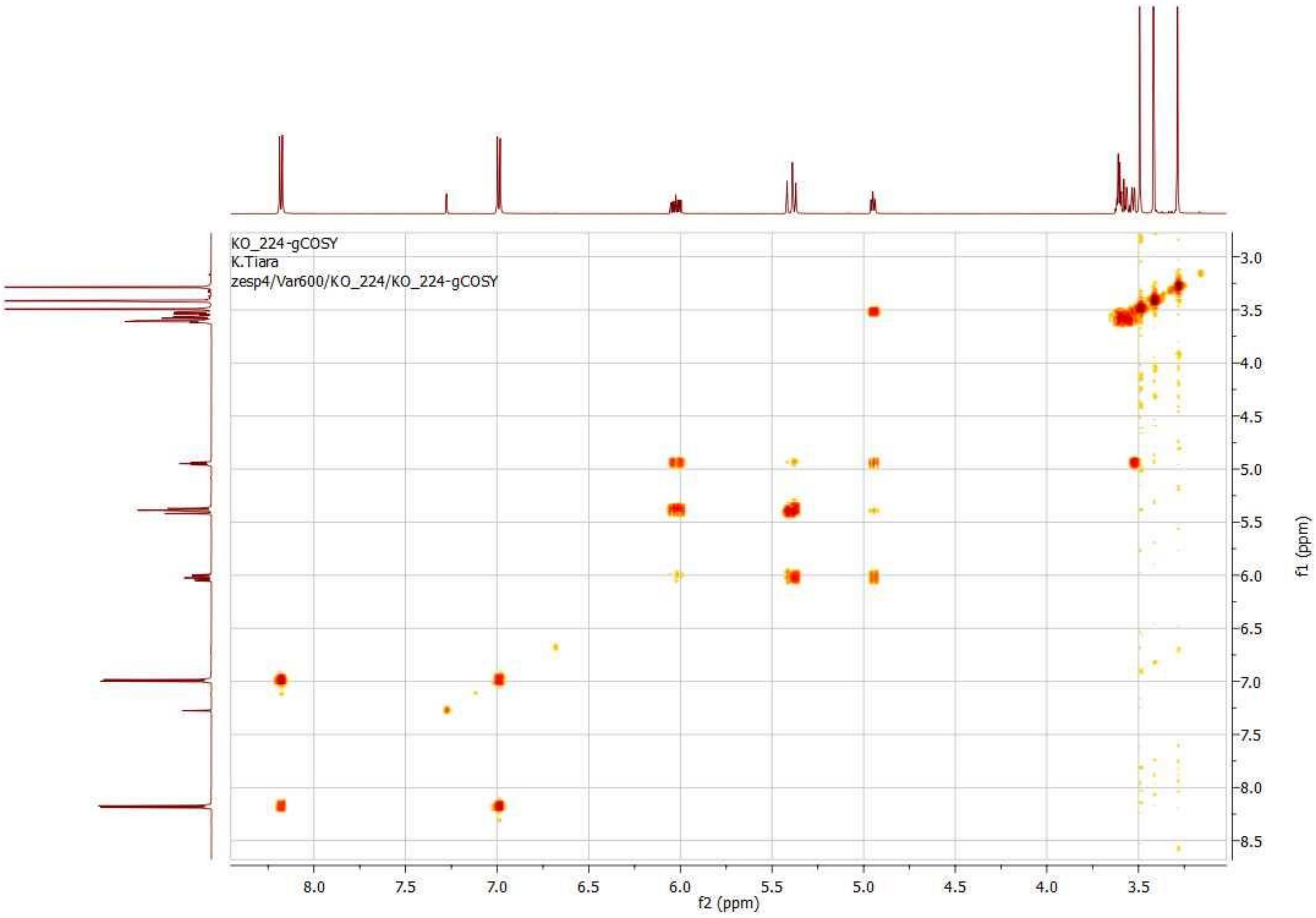

Figure S3 gCOSY spectrum of compound **14**.

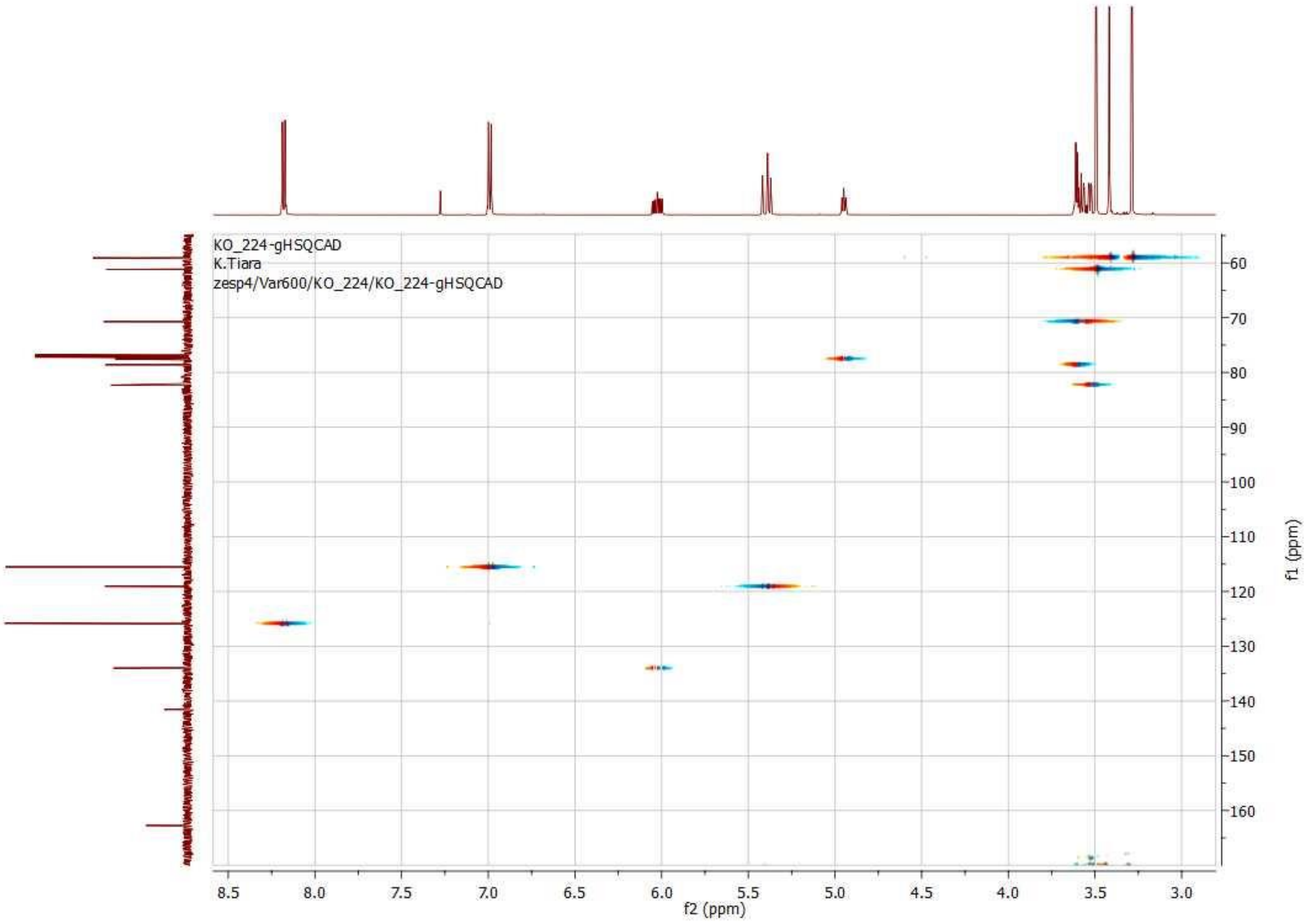

Figure S4 gHSQCAD spectrum of compound **14**.

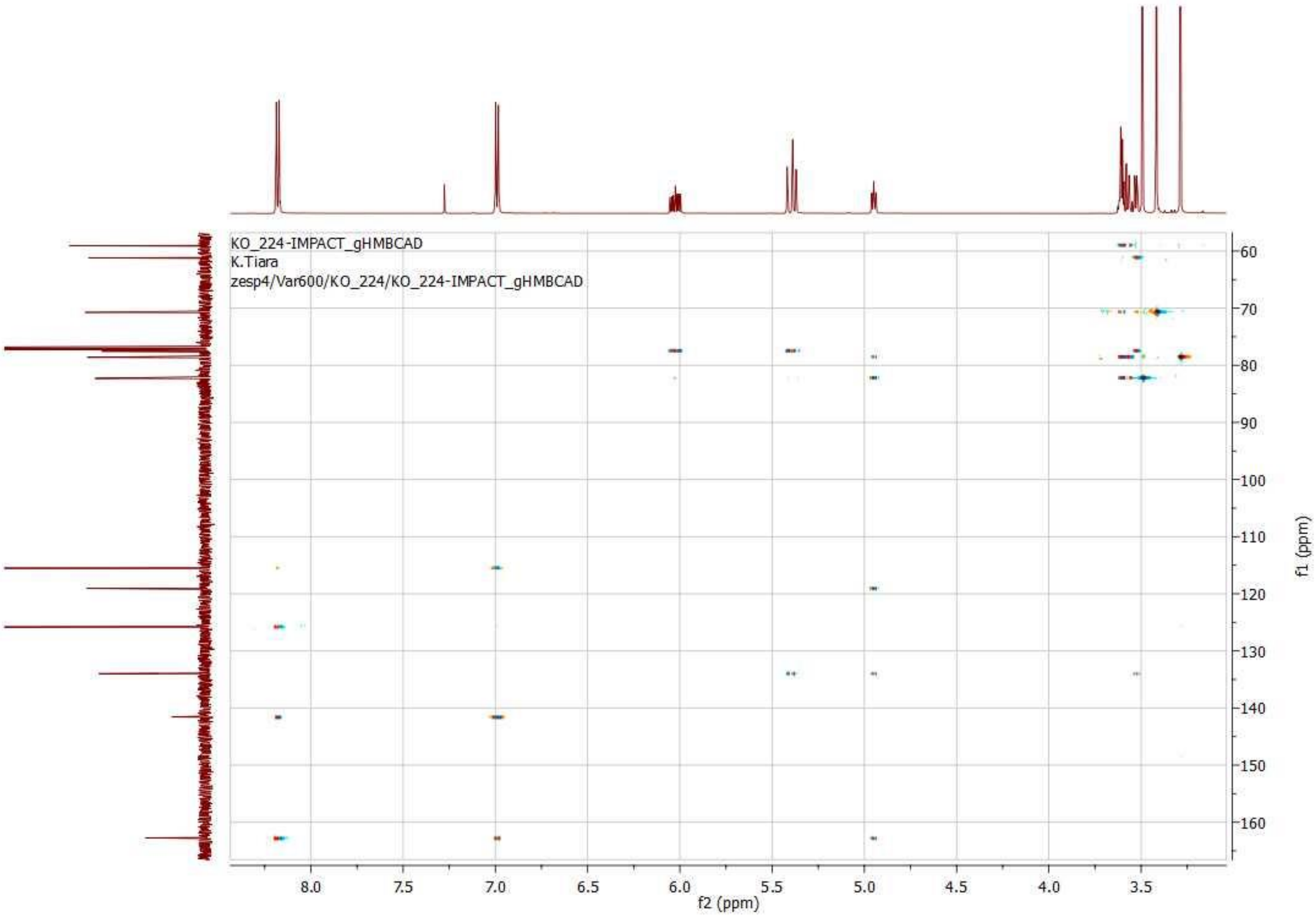

Figure S5 gHMBCAD spectrum of compound **14**.

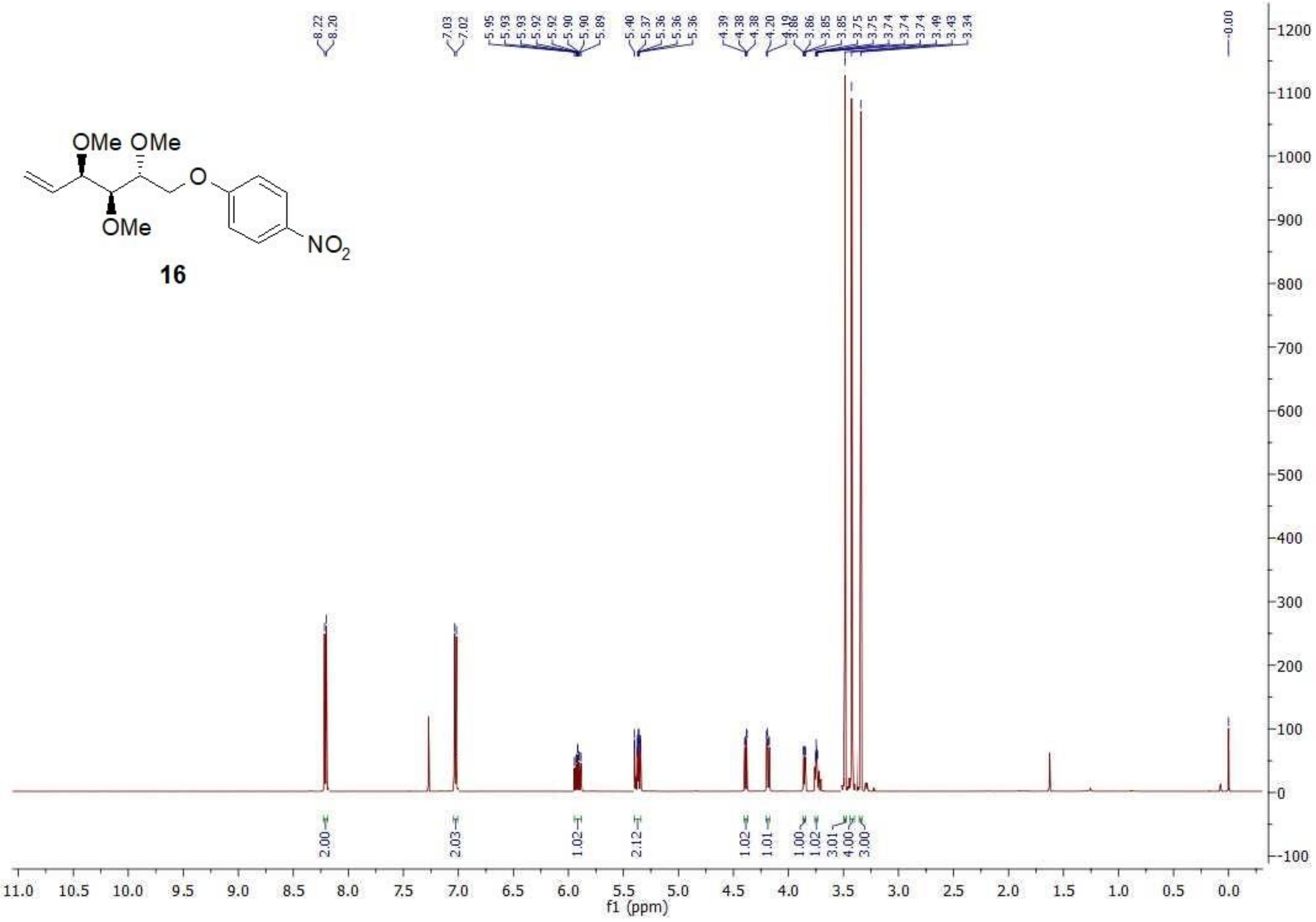

Figure S6 ^{1}H NMR spectrum of compound **16**.

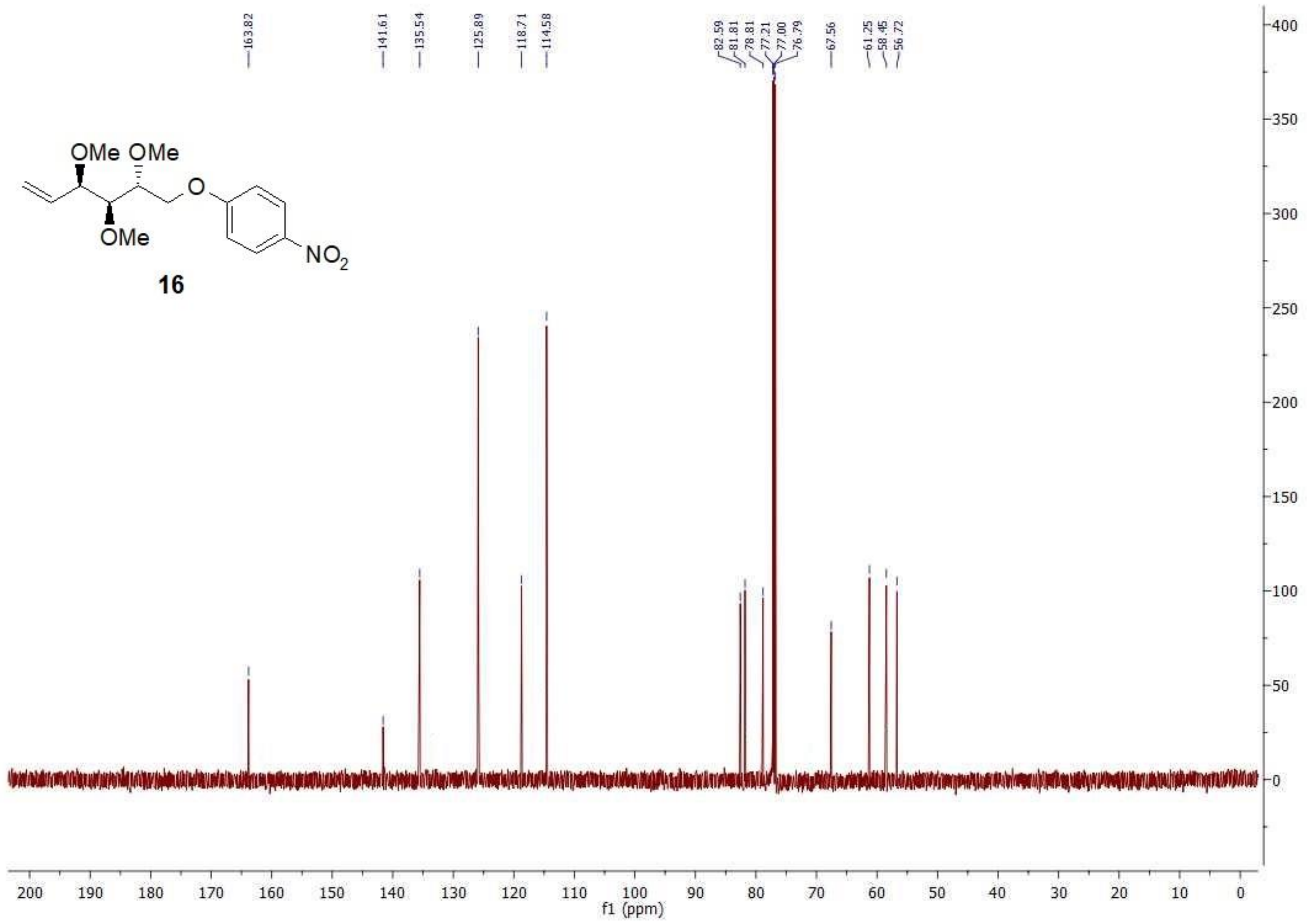

Figure S7 ^{13}C NMR spectrum of compound **16**.

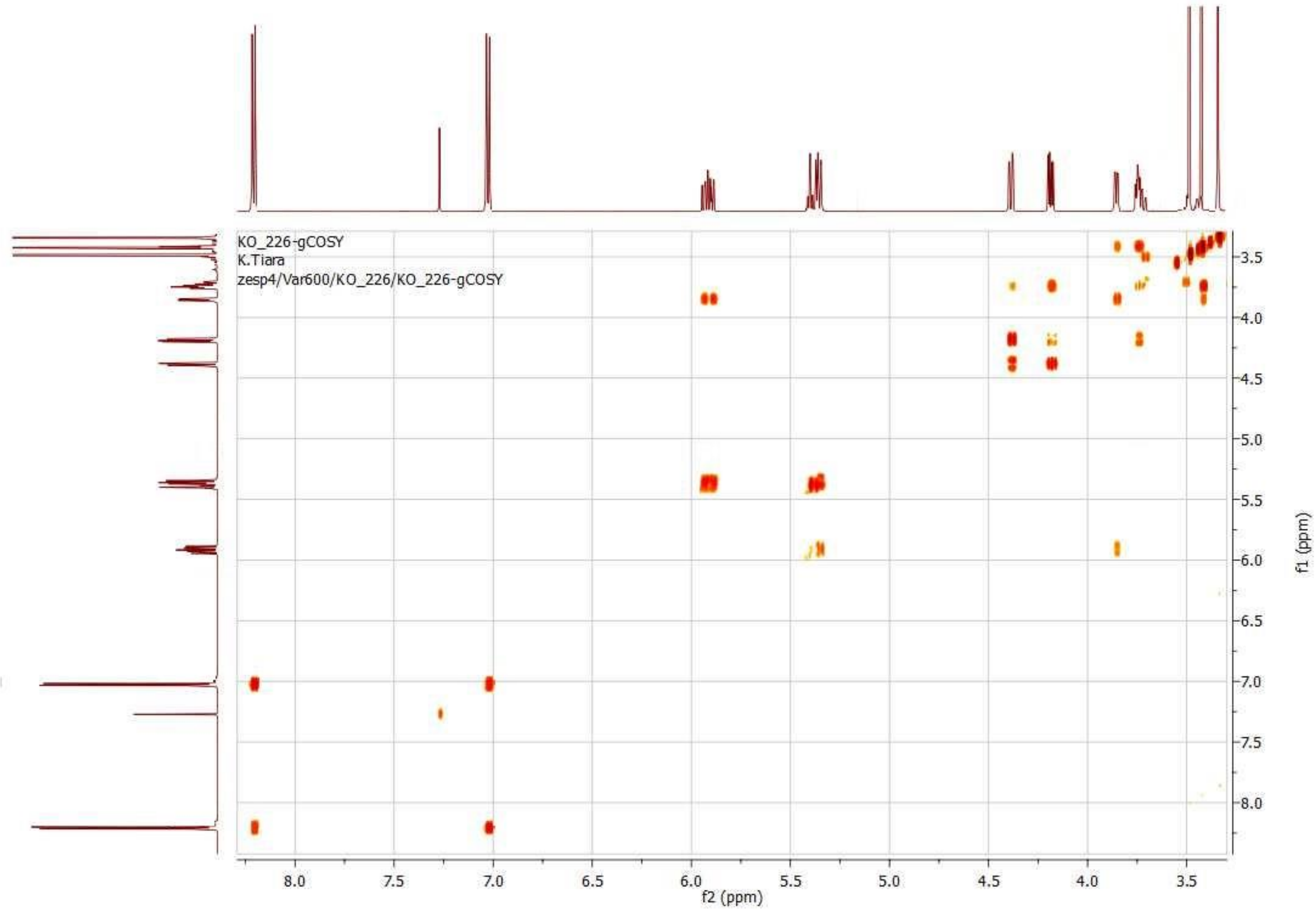

Figure S8 gCOSY spectrum of compound **16**.

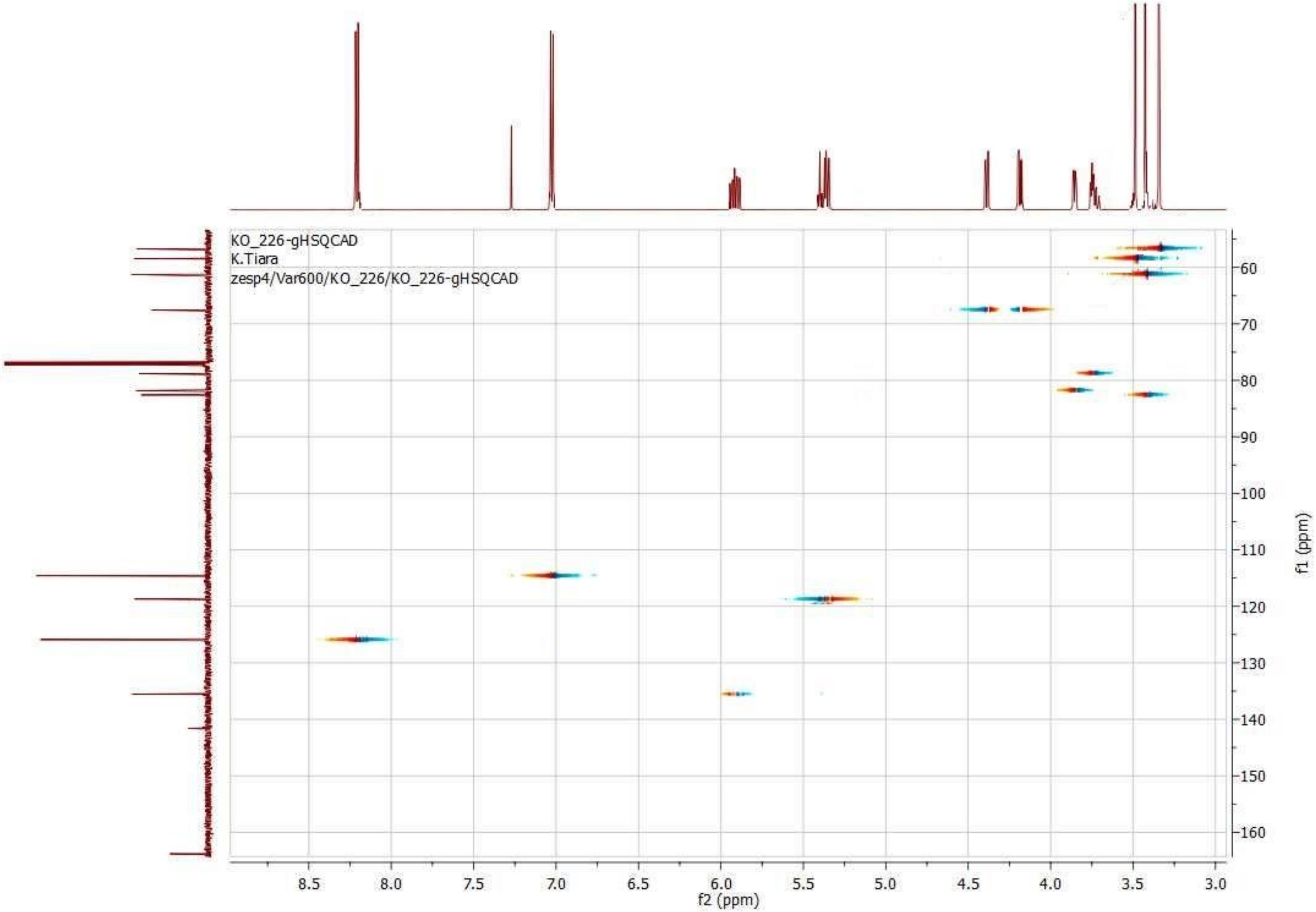

Figure S9 gHSQCAD spectrum of compound **16**.

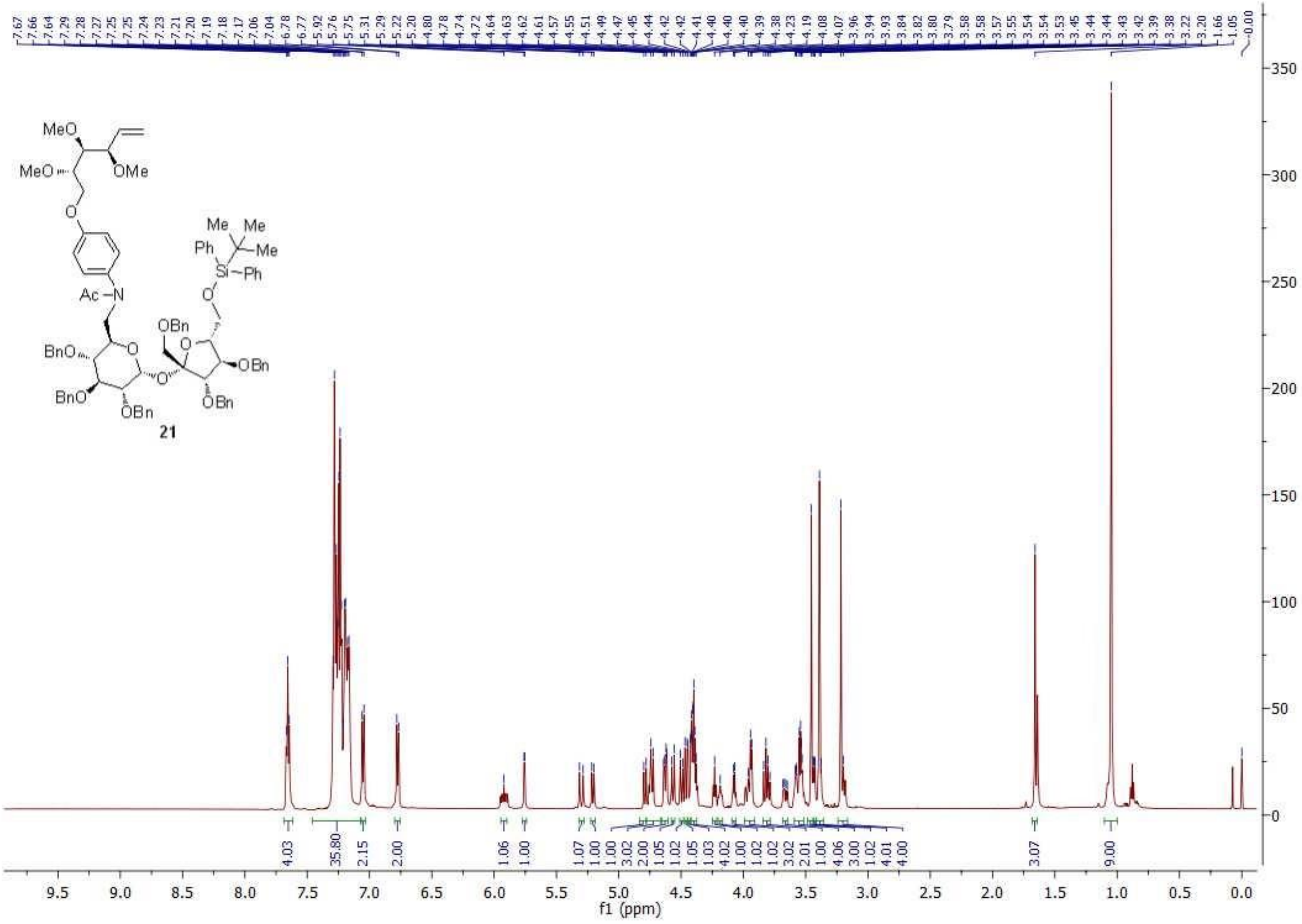

Figure S10 ^{1}H NMR spectrum of compound **21**.

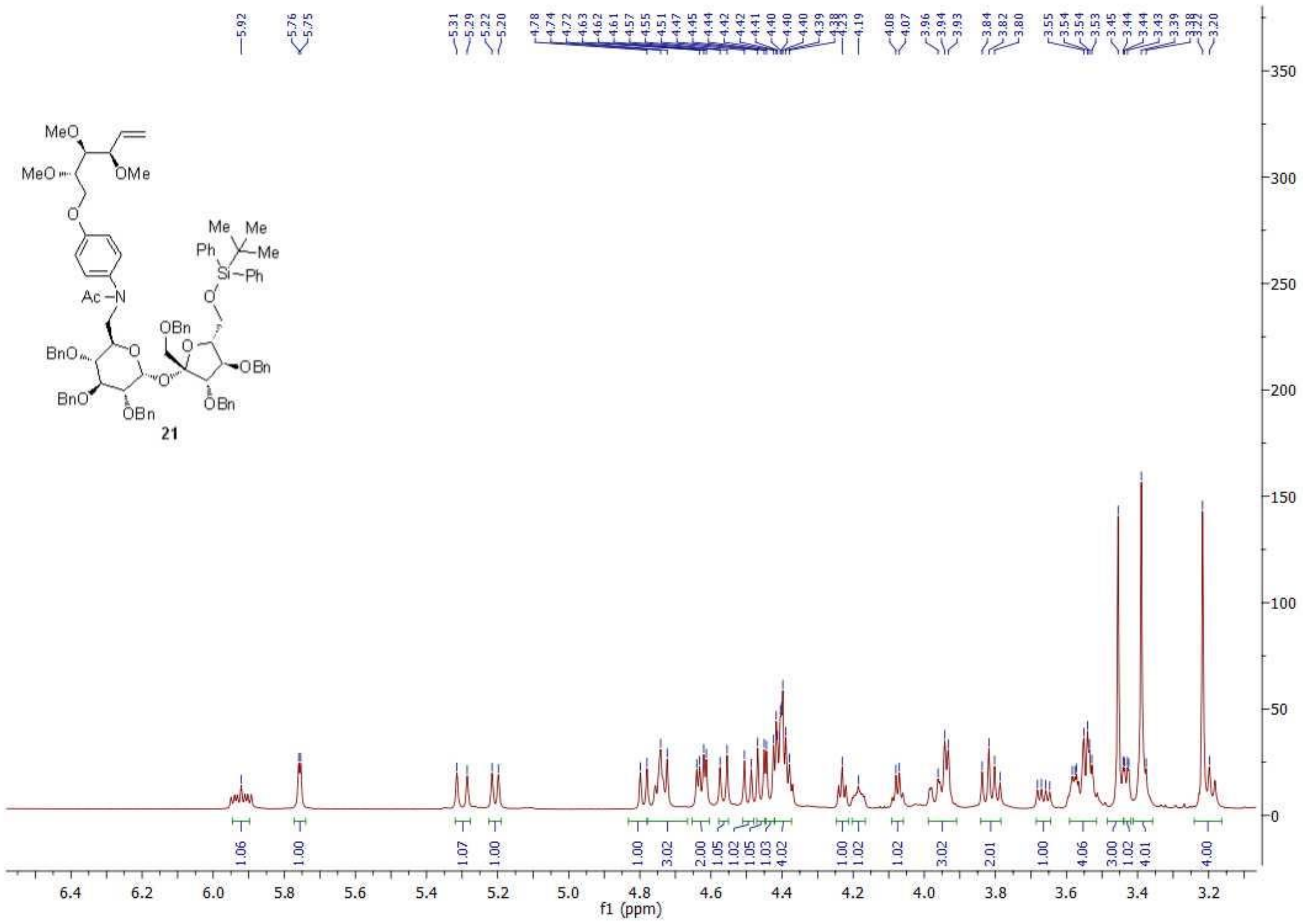

Figure S11 ^{1}H NMR spectrum (aliphatic part) of compound **21**.

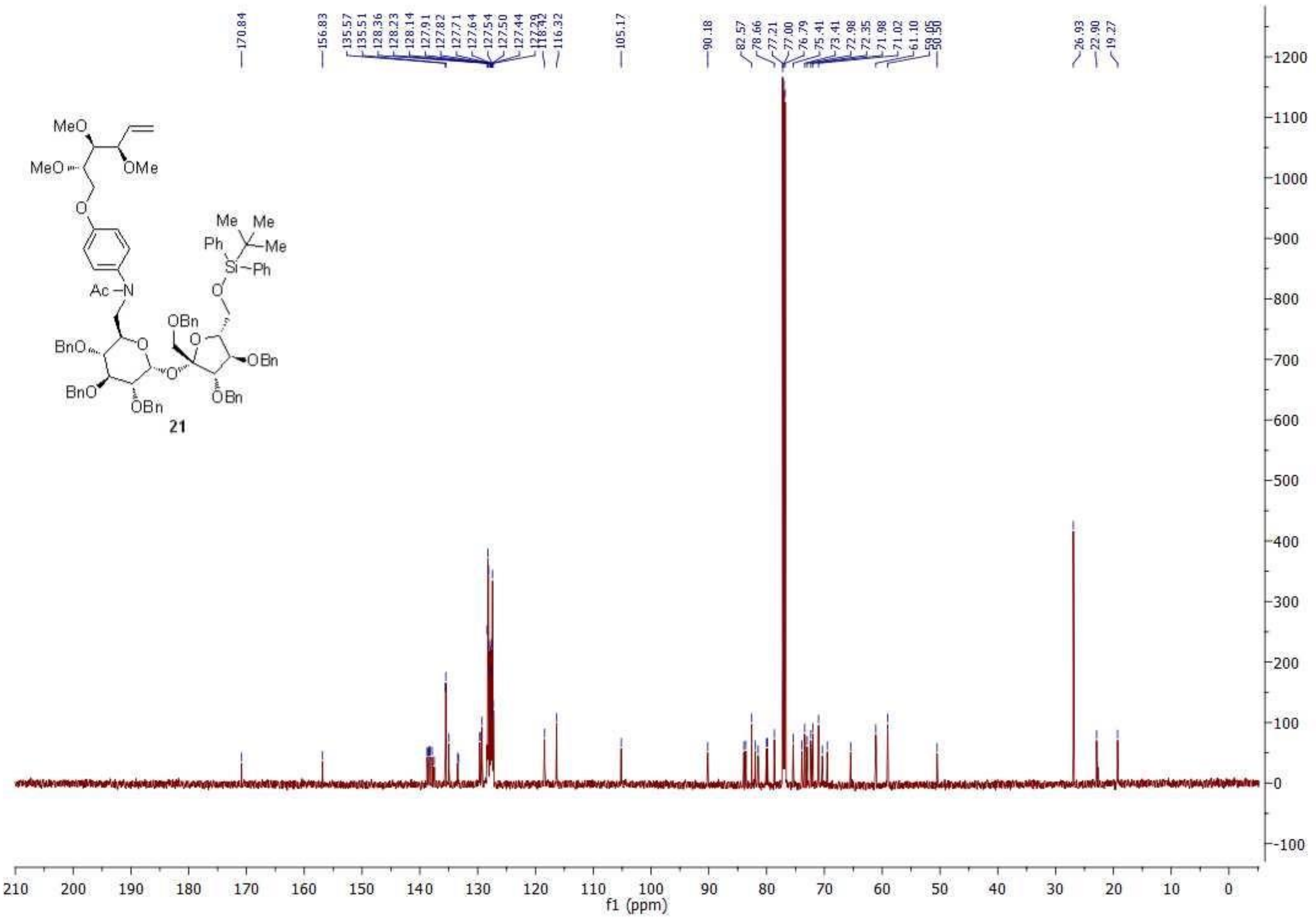

Figure S12 ^{13}C NMR spectrum of compound **21**.

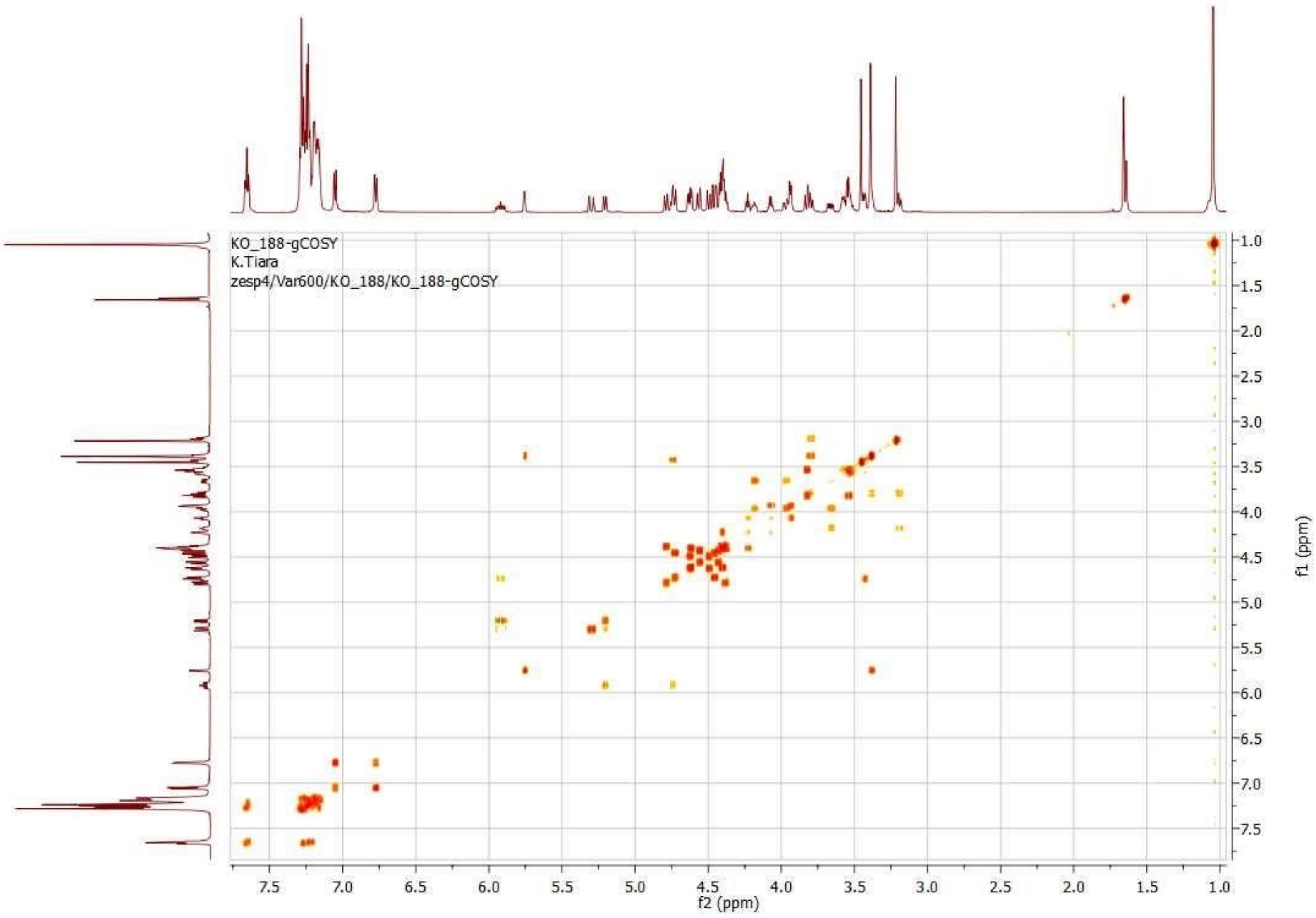

Figure S13 COSY spectrum of compound **21**.

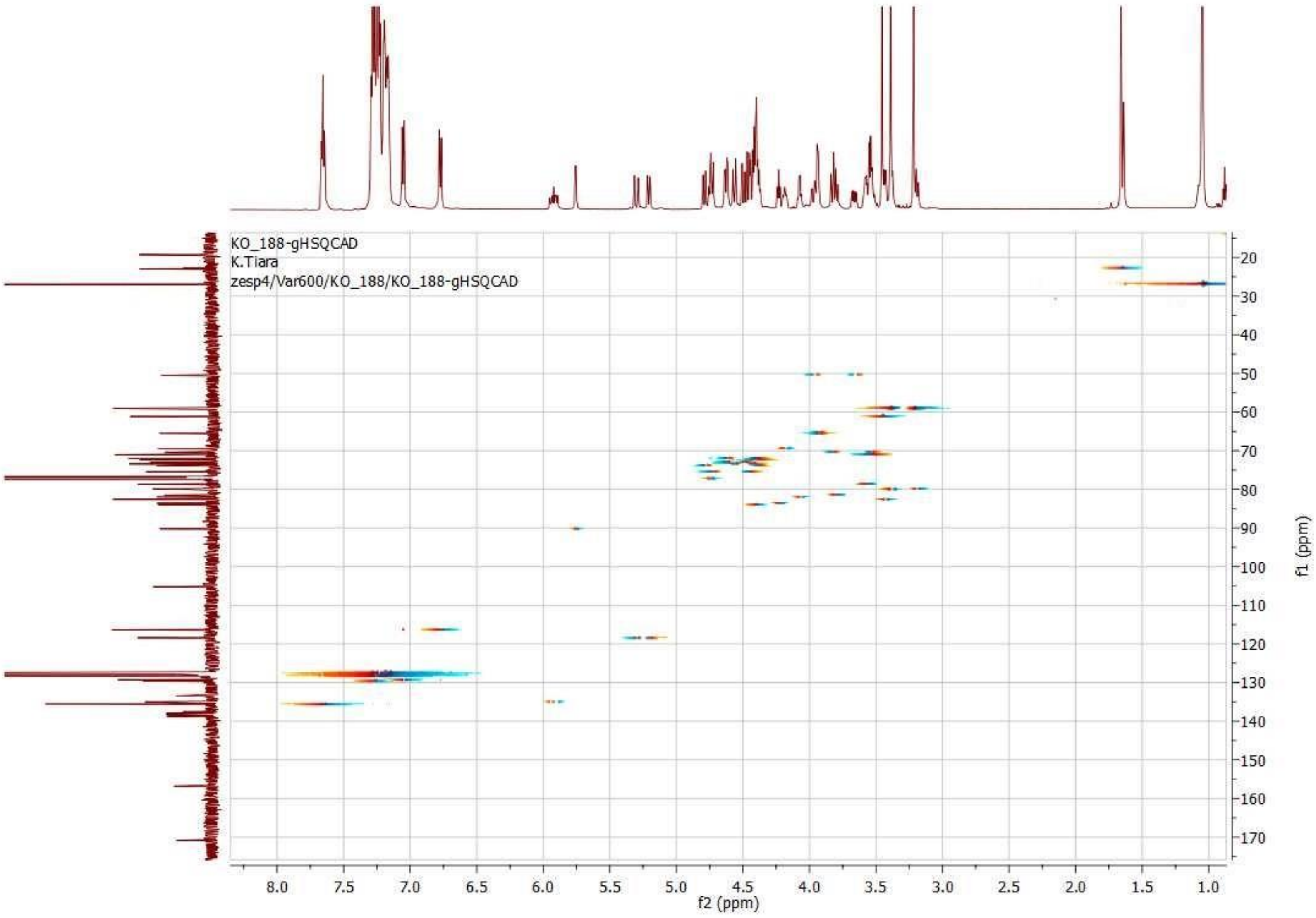

Figure S14 gHSQCAD spectrum of compound **21**.

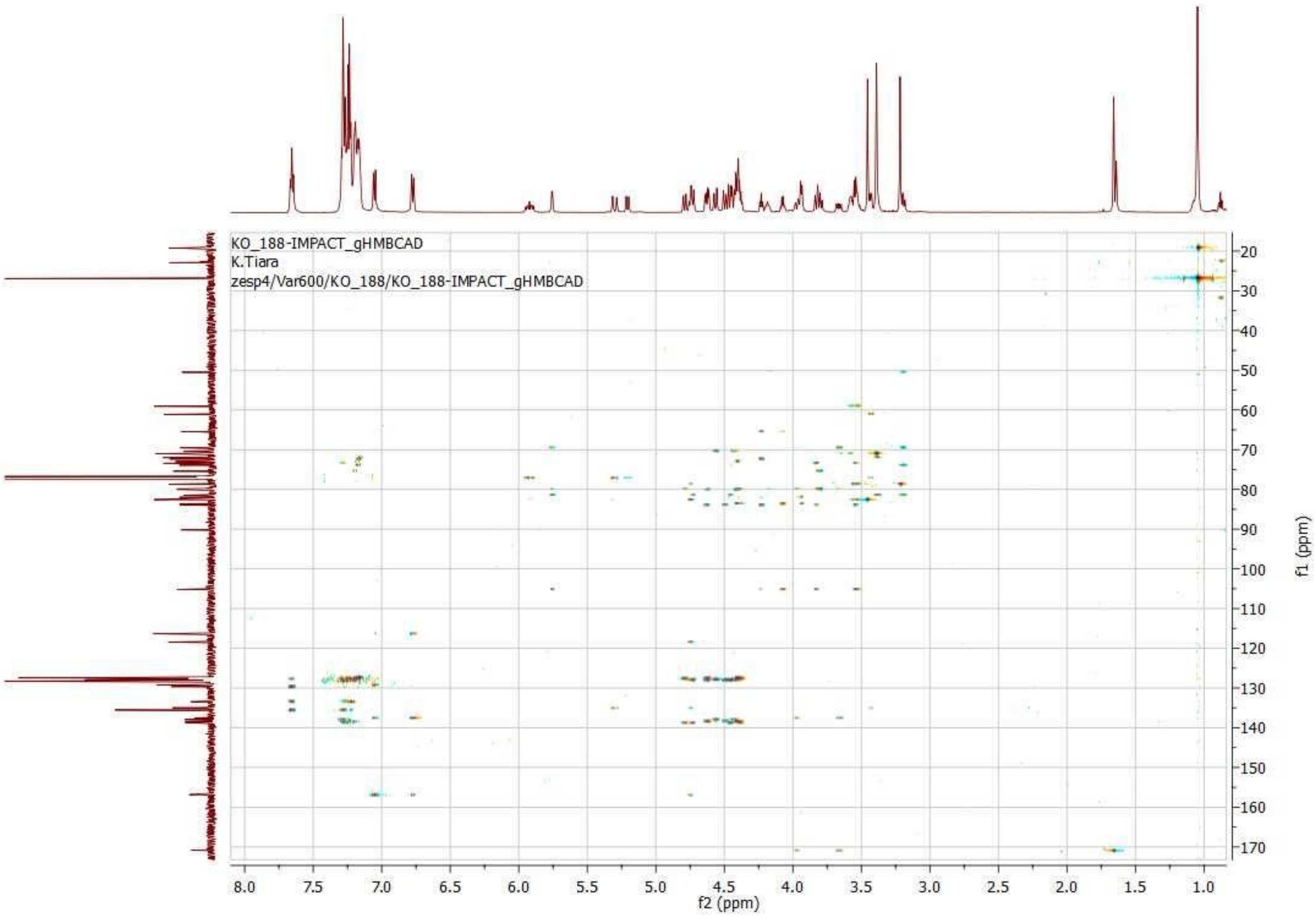

Figure S15 gHMBCAD spectrum of compound **21**.

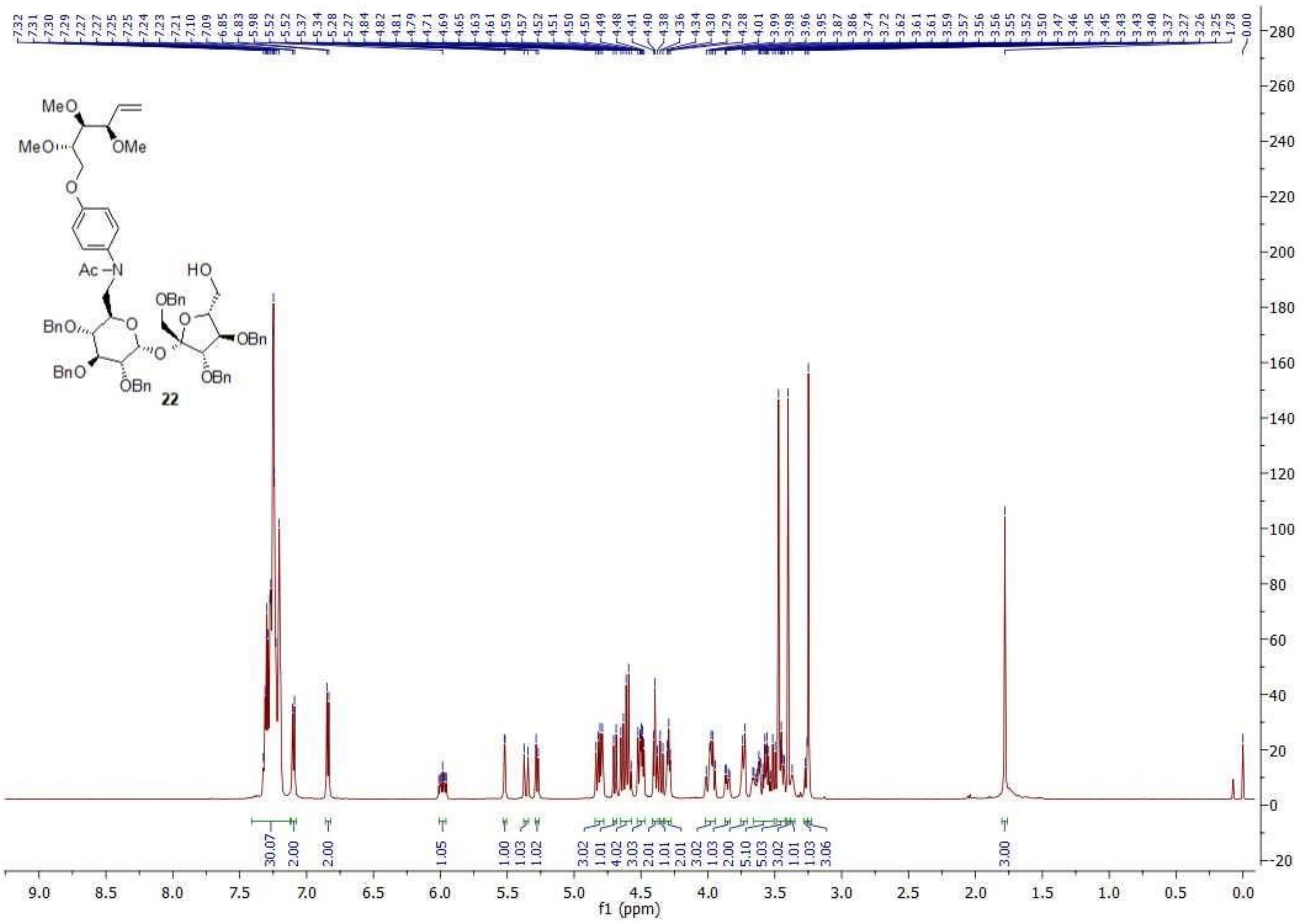

Figure S16 ^{1}H NMR spectrum of compound **22**.

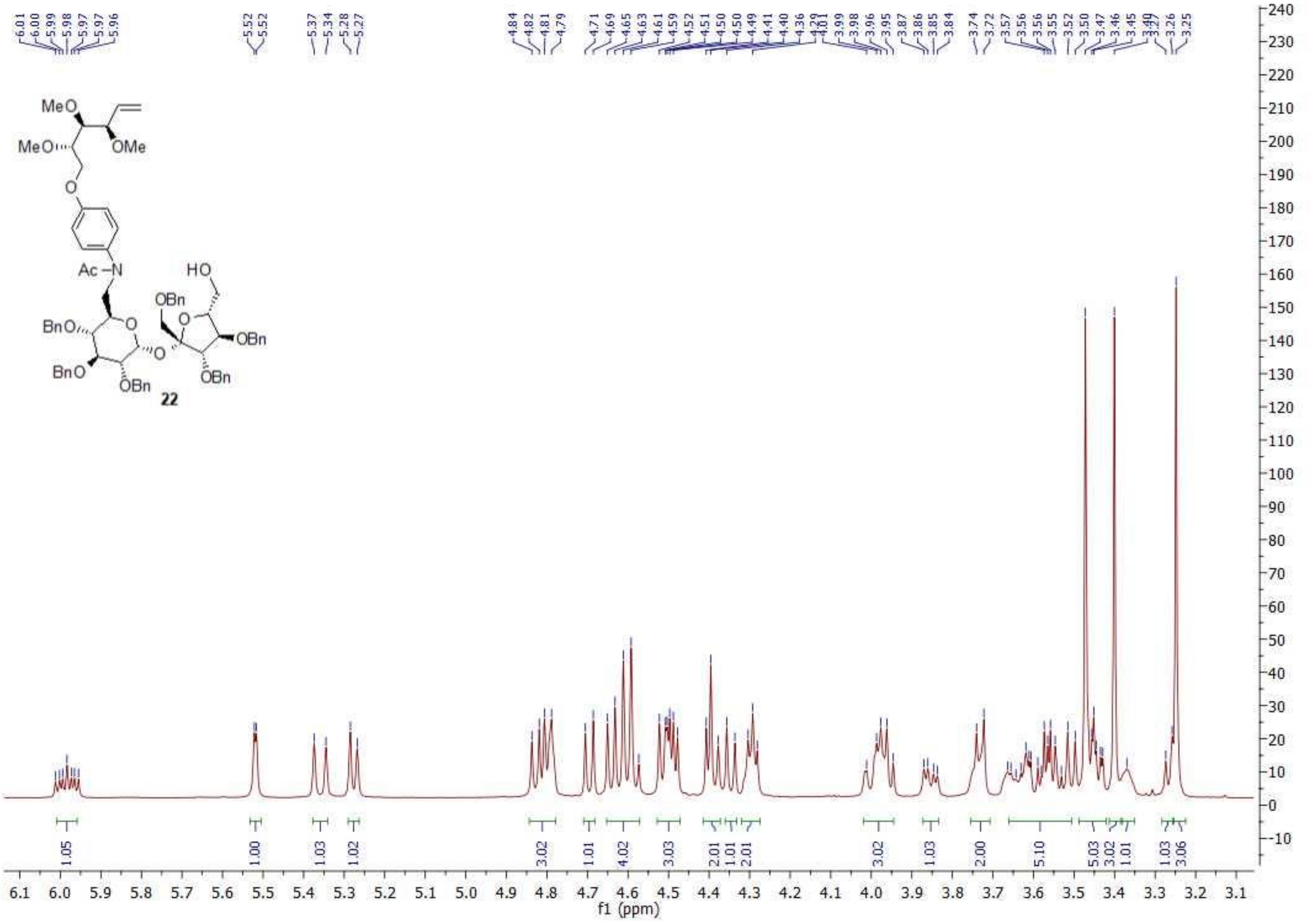

Figure S17 ^{1}H NMR spectrum (aliphatic part) of compound **22**.

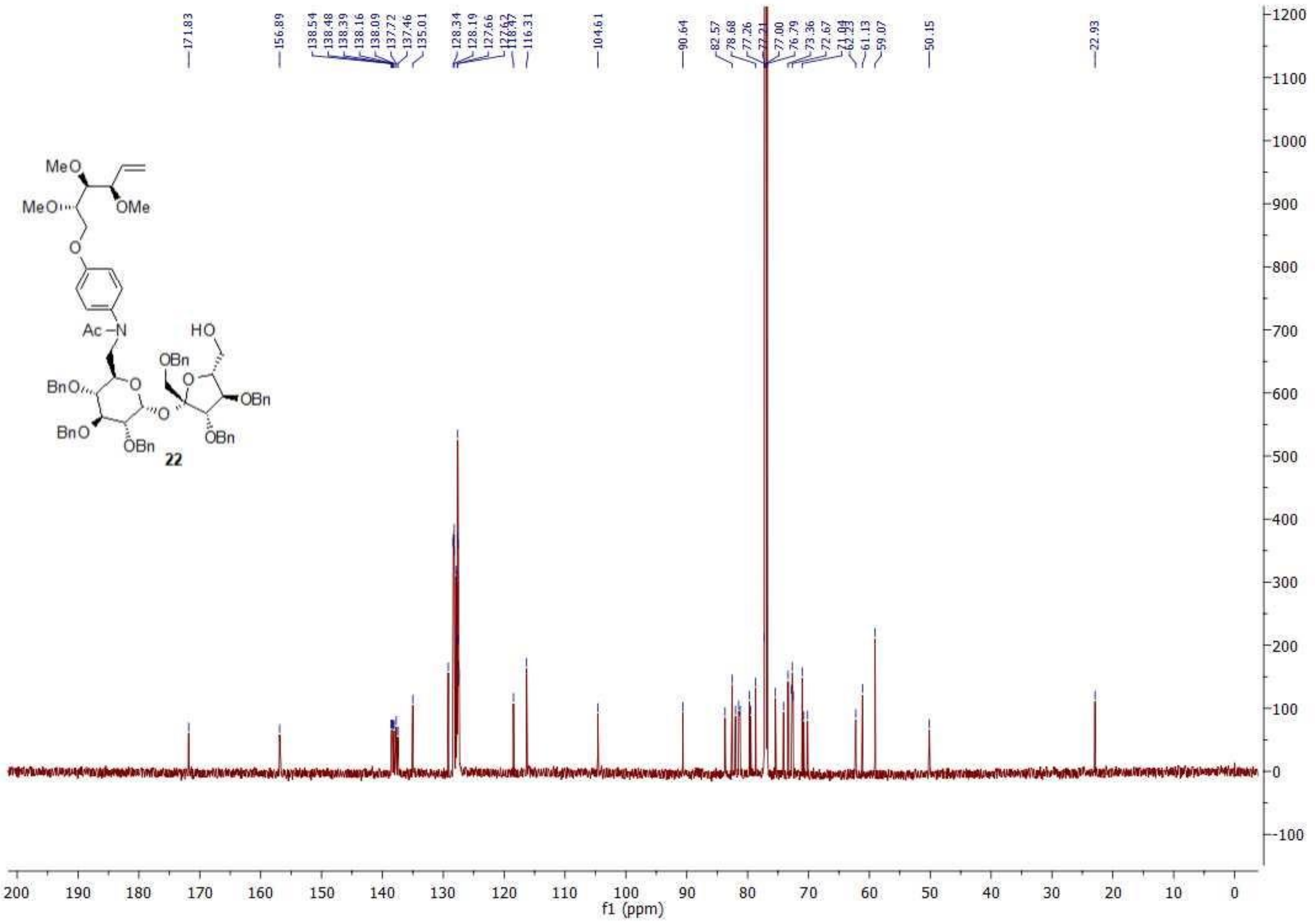

Figure S18 ^{13}C NMR spectrum of compound **22**.

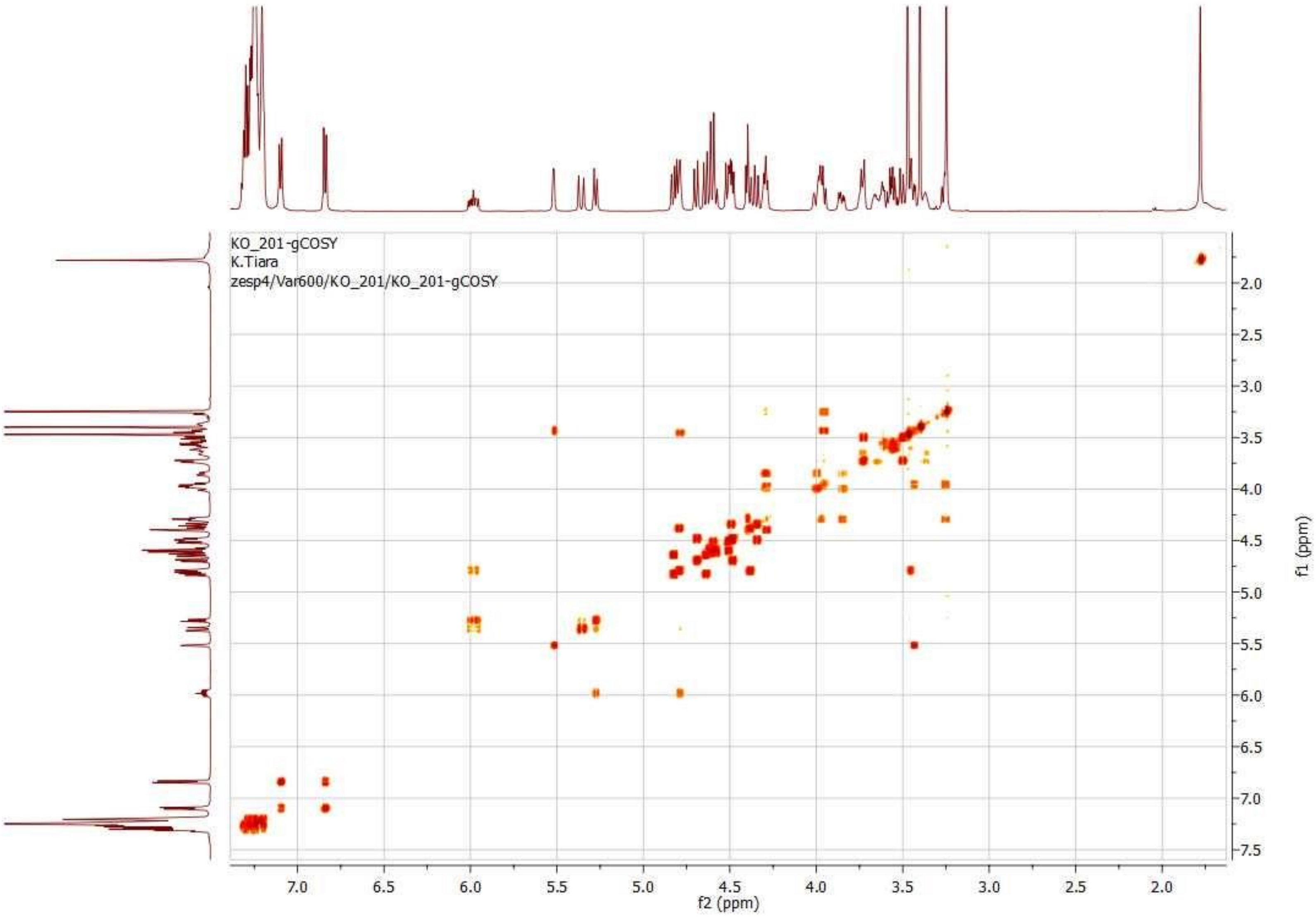

Figure S19 gCOSY spectrum of compound **22**.

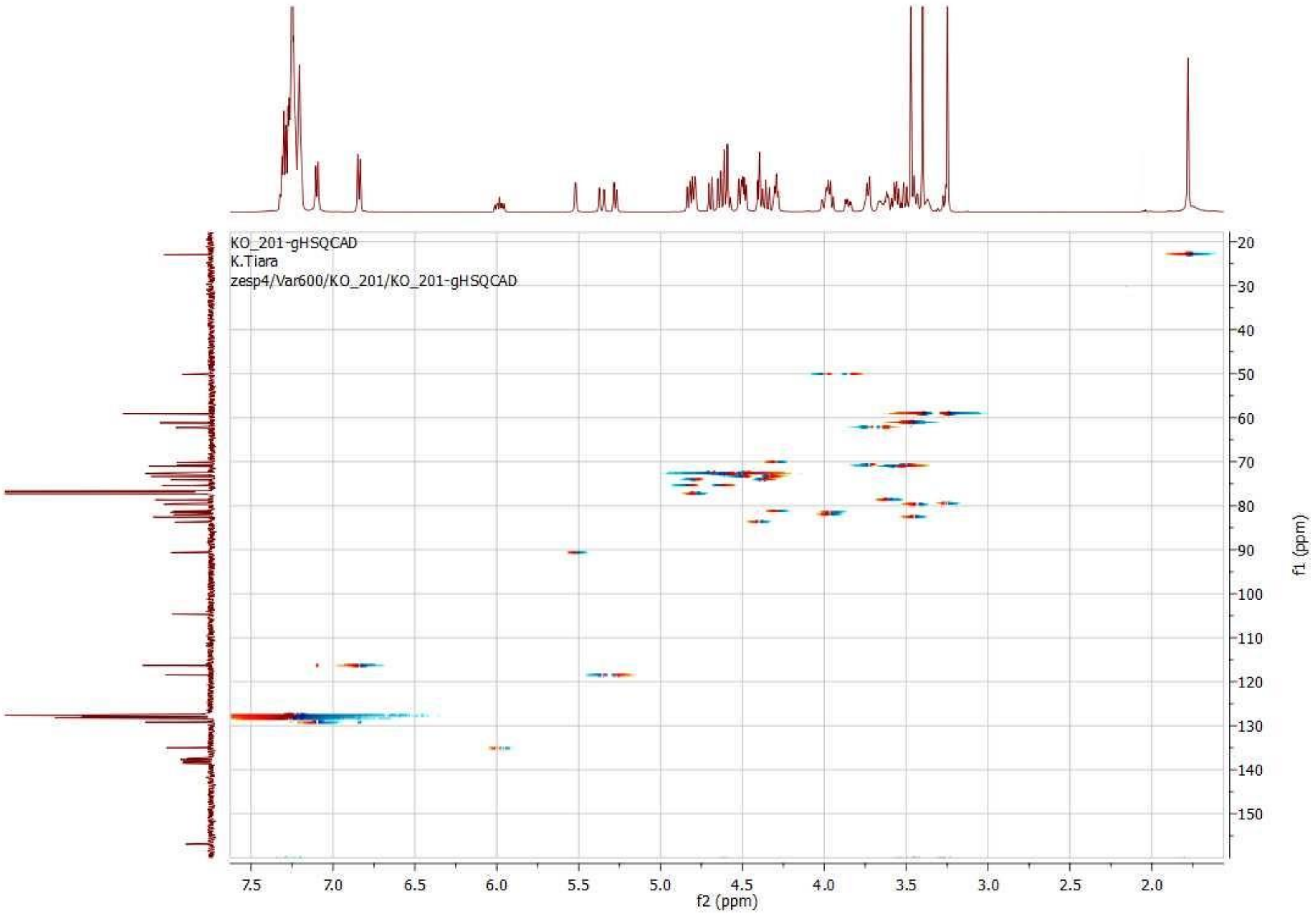

Figure S20 gHSQCAD spectrum of compound **22**.

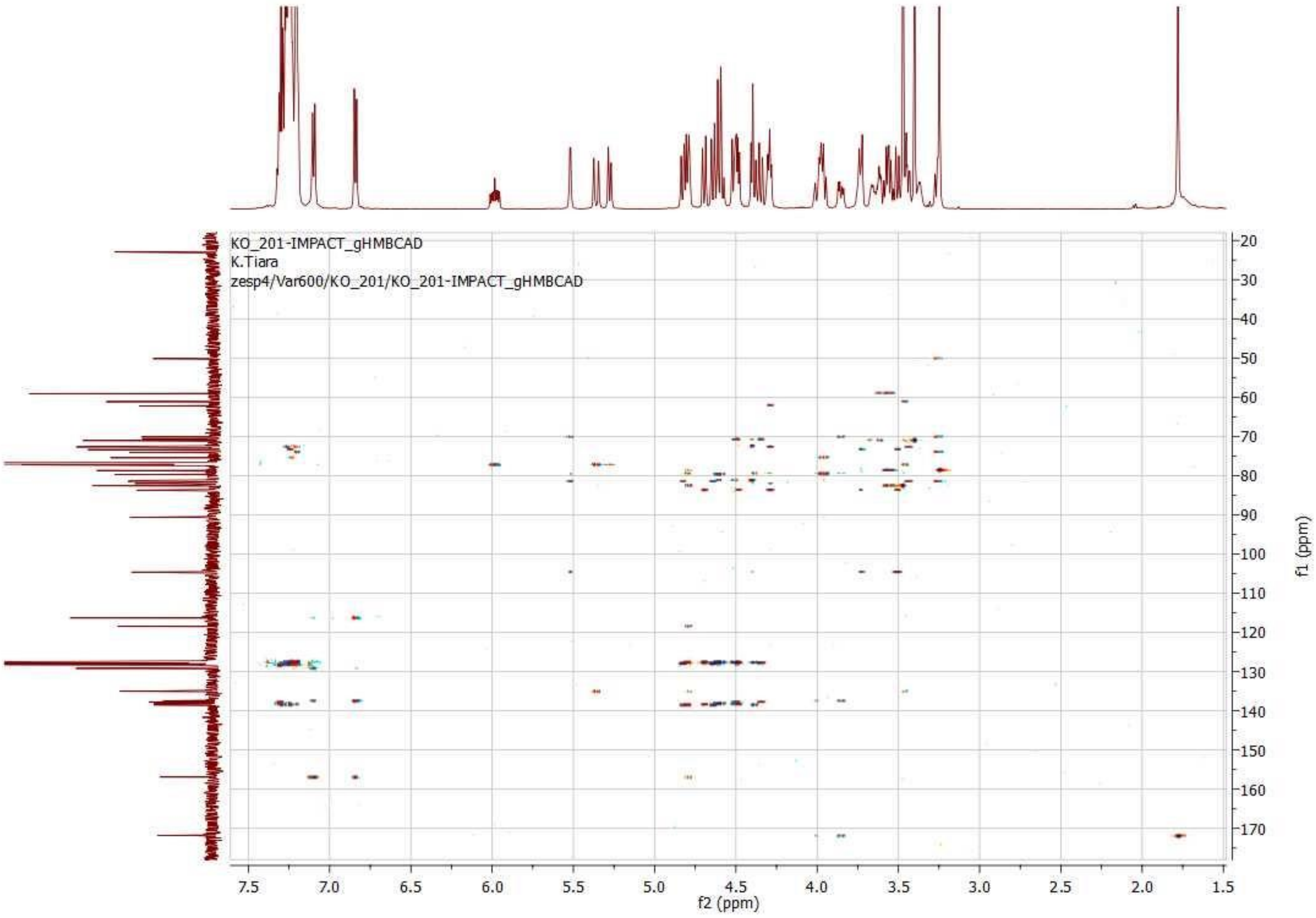

Figure S21 gHMBCAD spectrum of compound **22**.

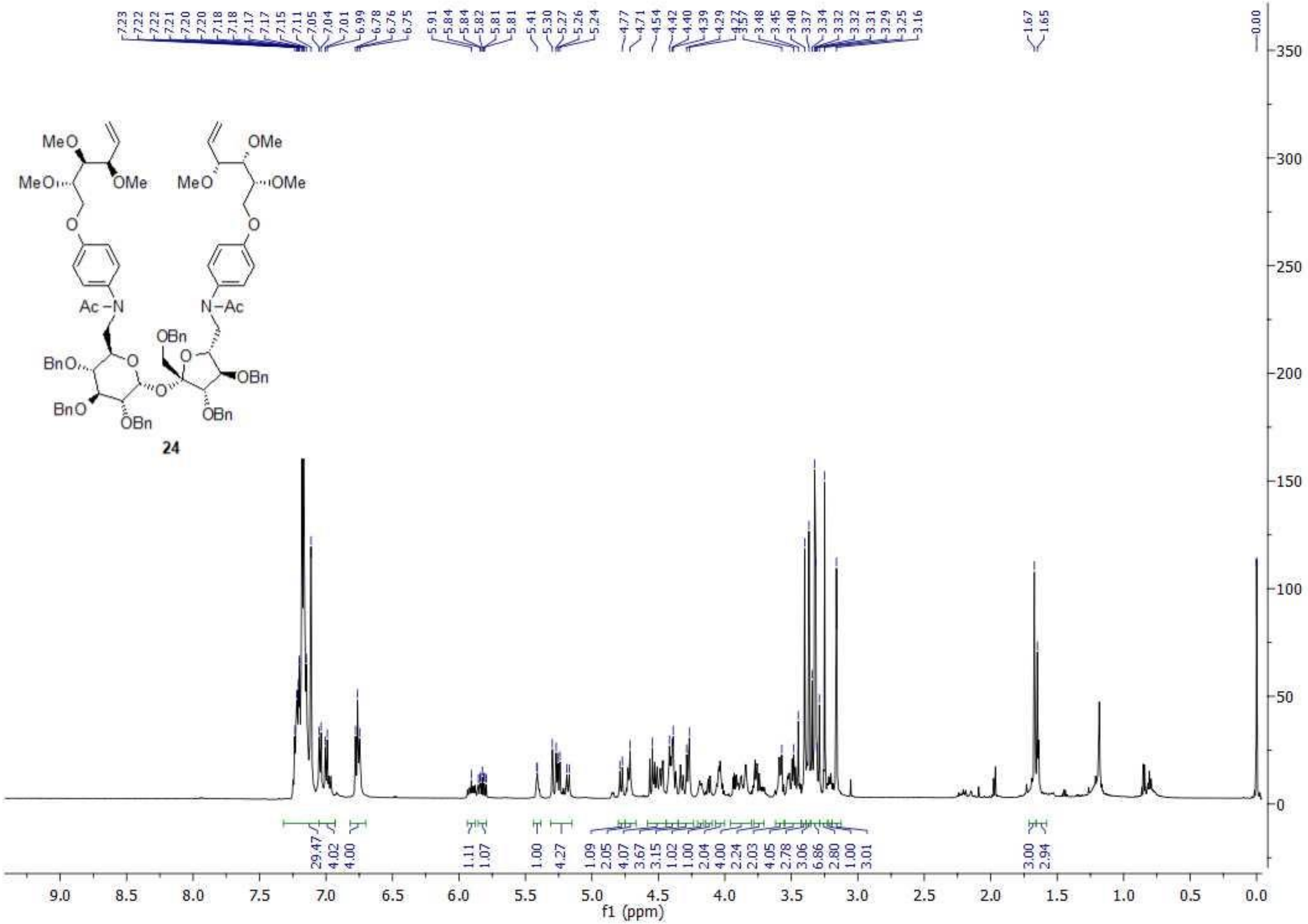

Figure S22 ^{1}H NMR spectrum of compound **24**.

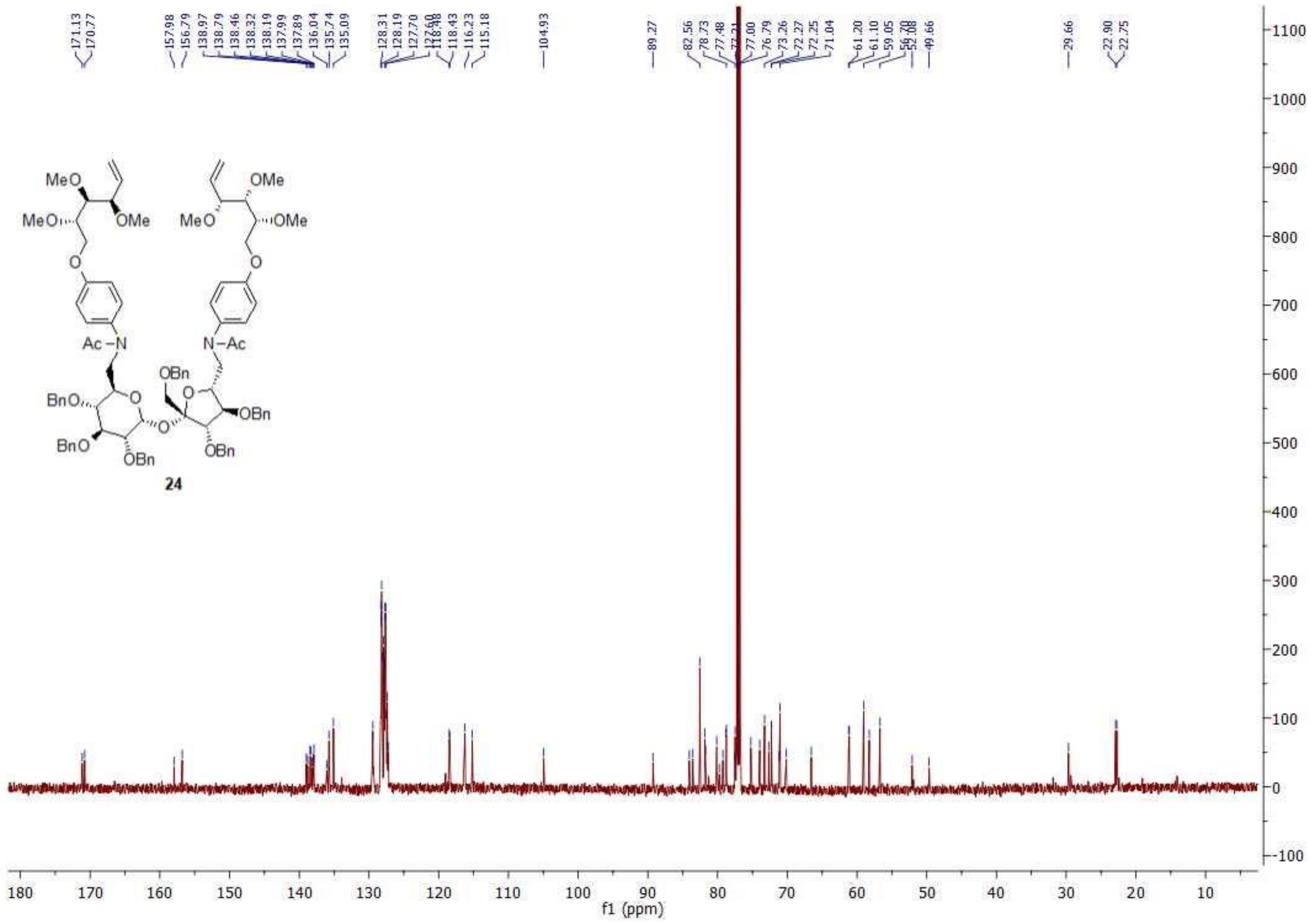

Figure S23 ^{13}C NMR spectrum of compound **24**.

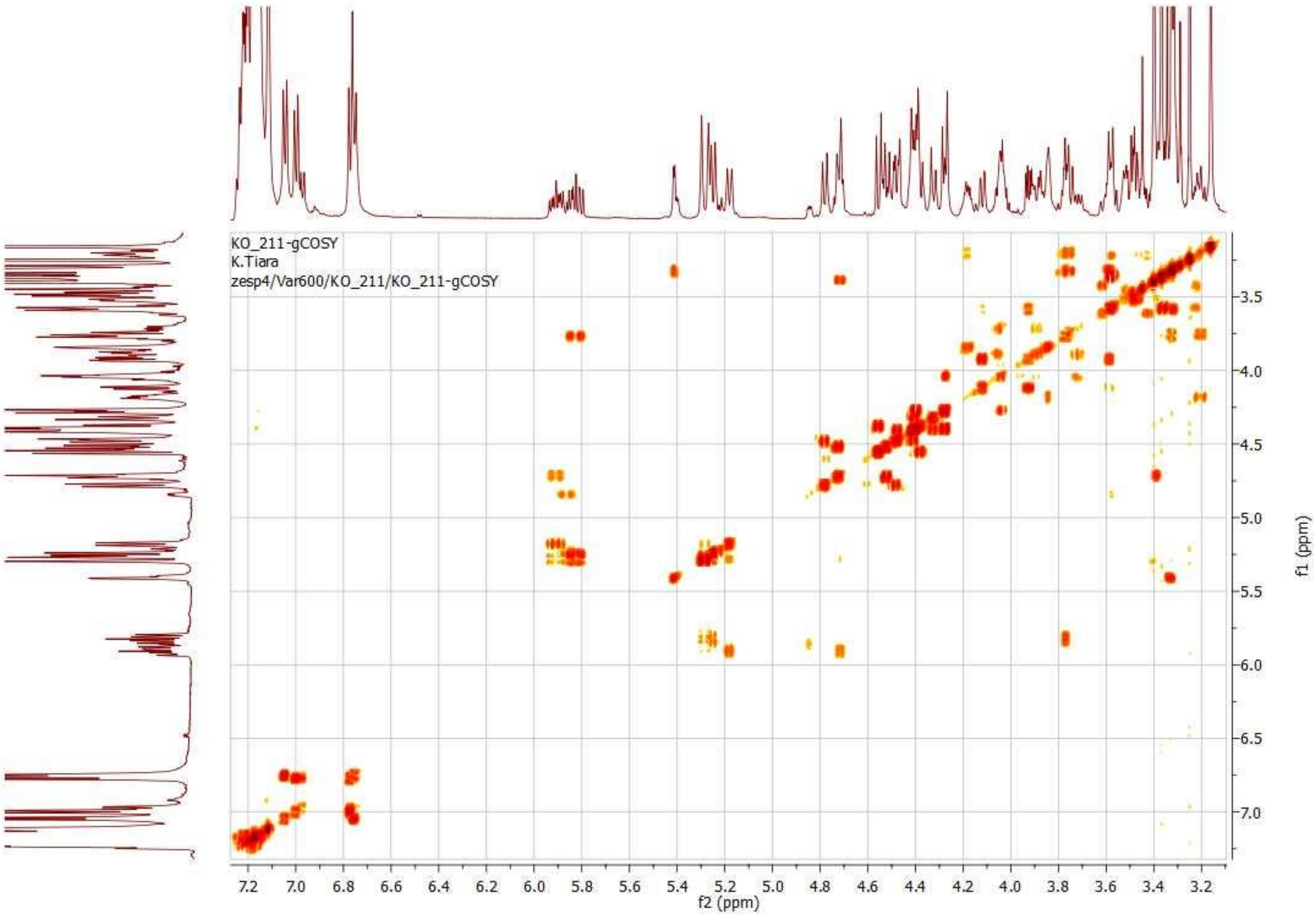

Figure S24 gCOSY NMR spectrum of compound **24**.

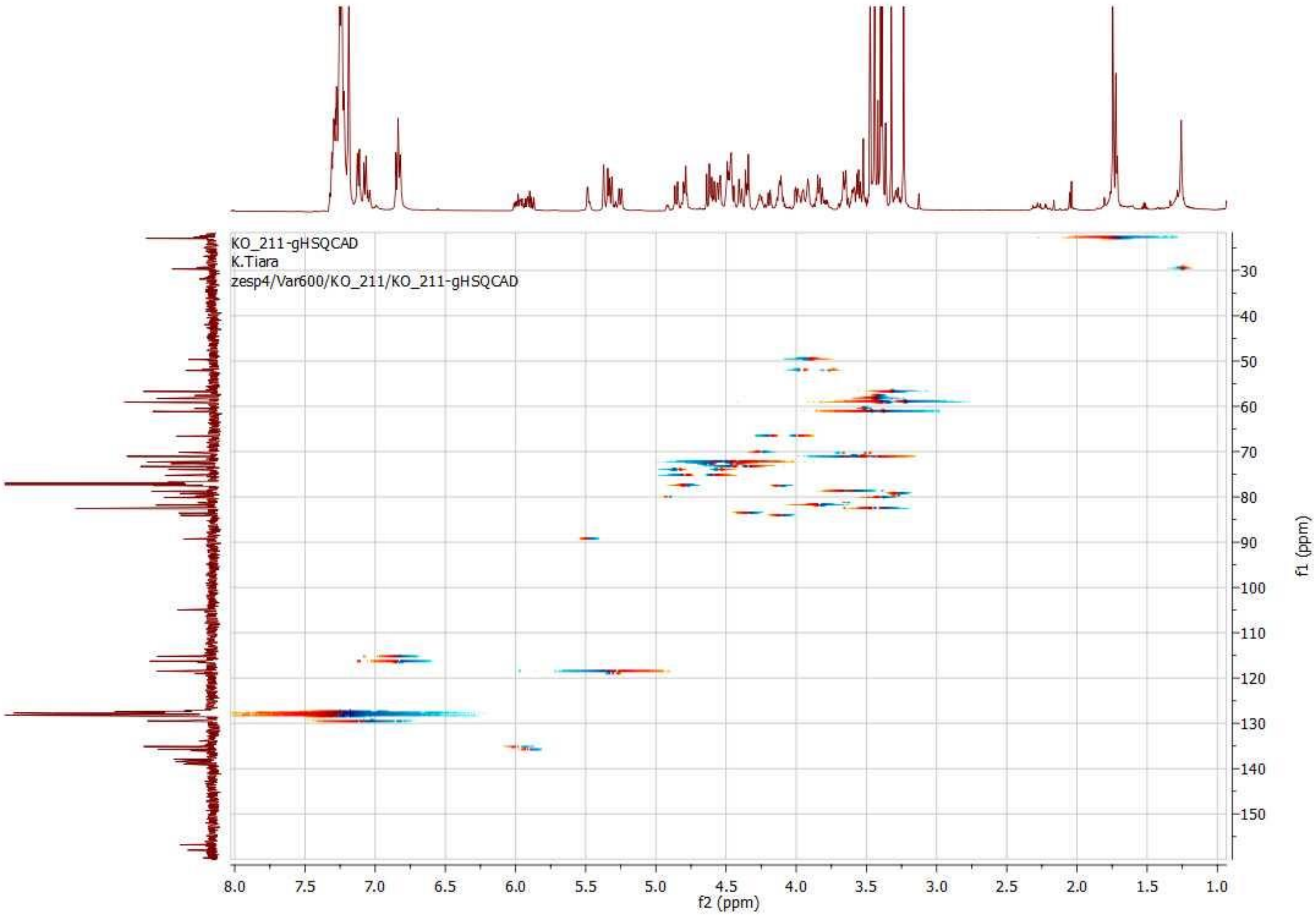

Figure S25 gHSQCAD NMR spectrum of compound **24**.

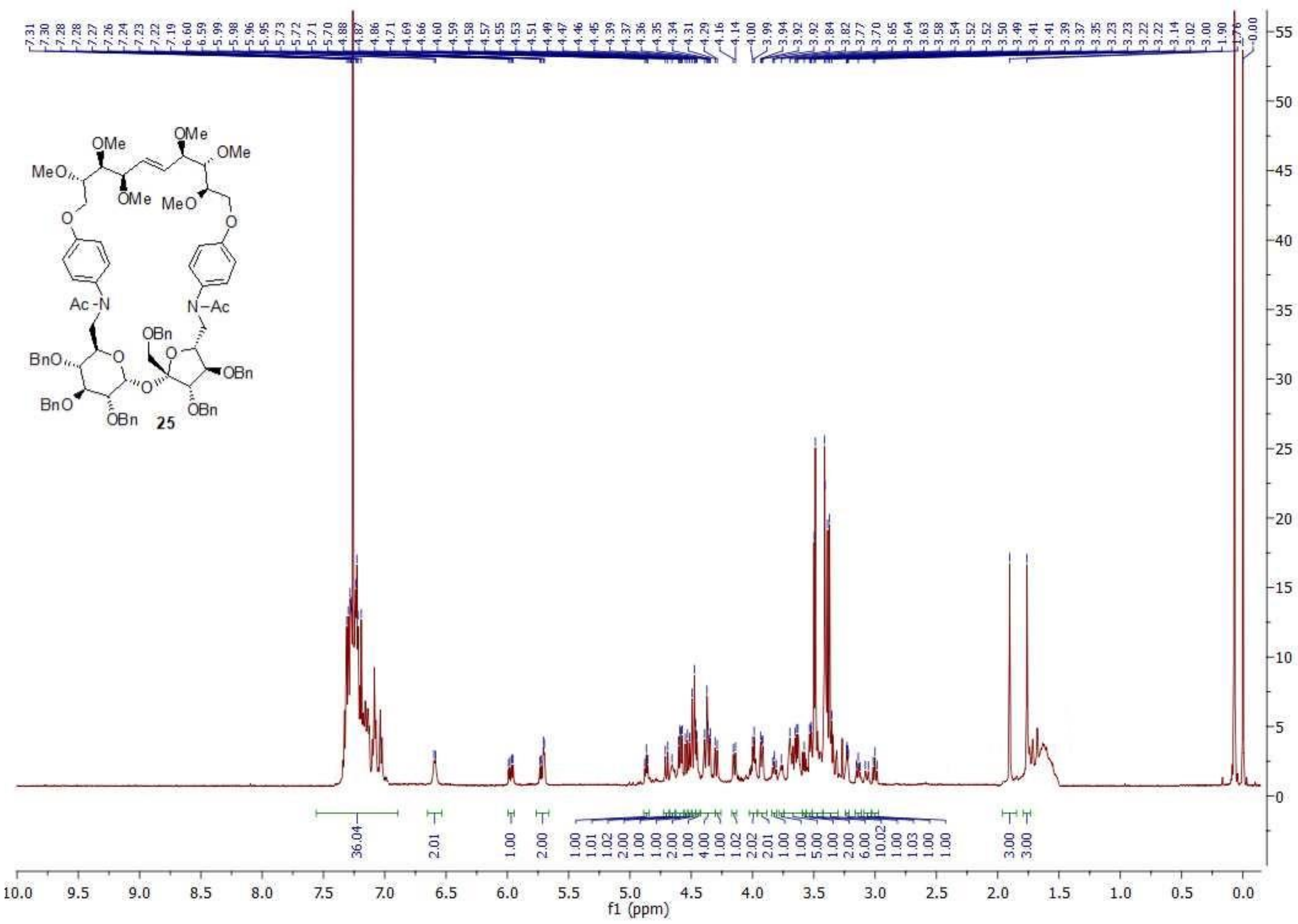

Figure S26 ^{1}H NMR spectrum of compound **25**.

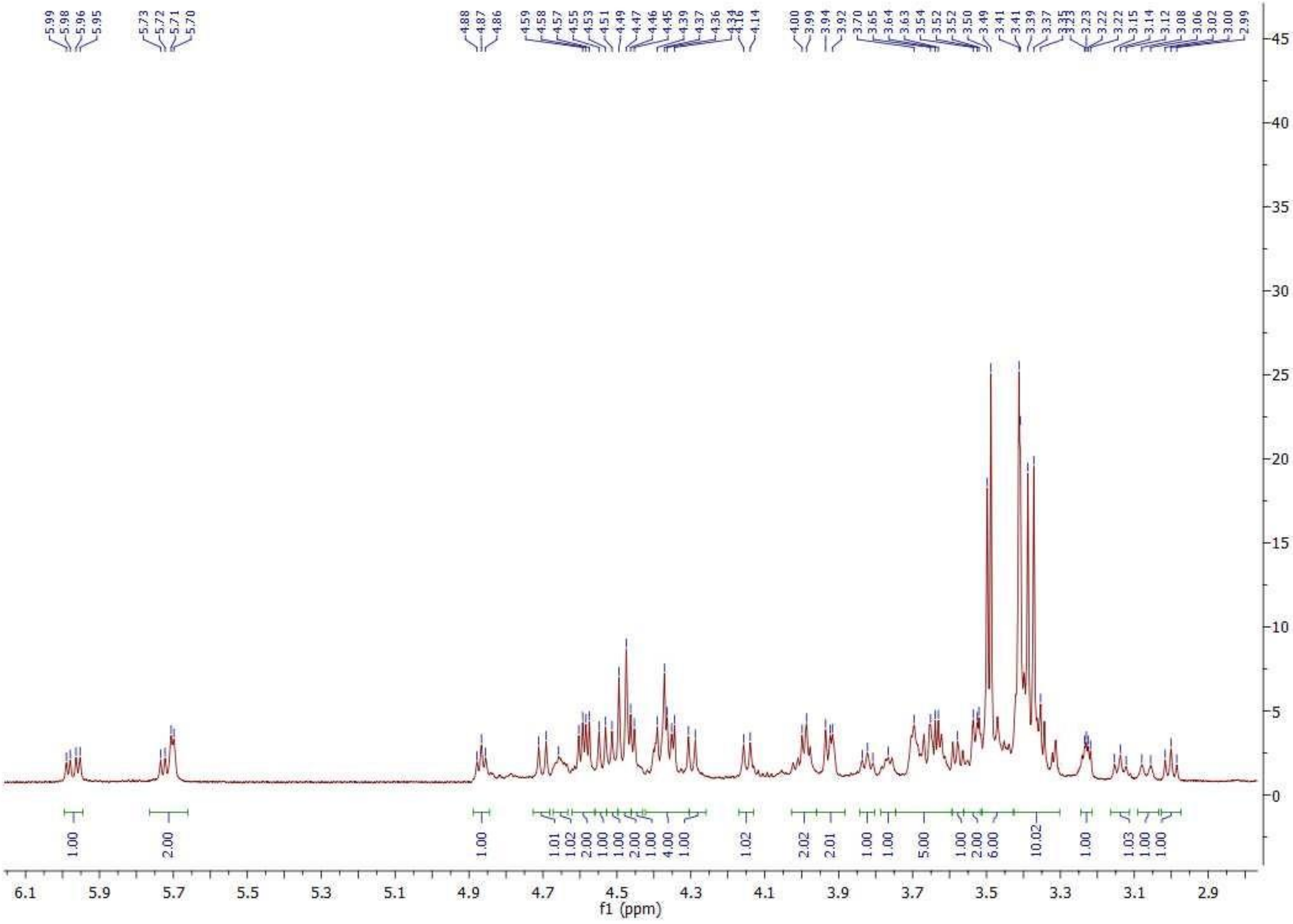

Figure S27 ^{1}H NMR spectrum (aliphatic part) of compound **25**.

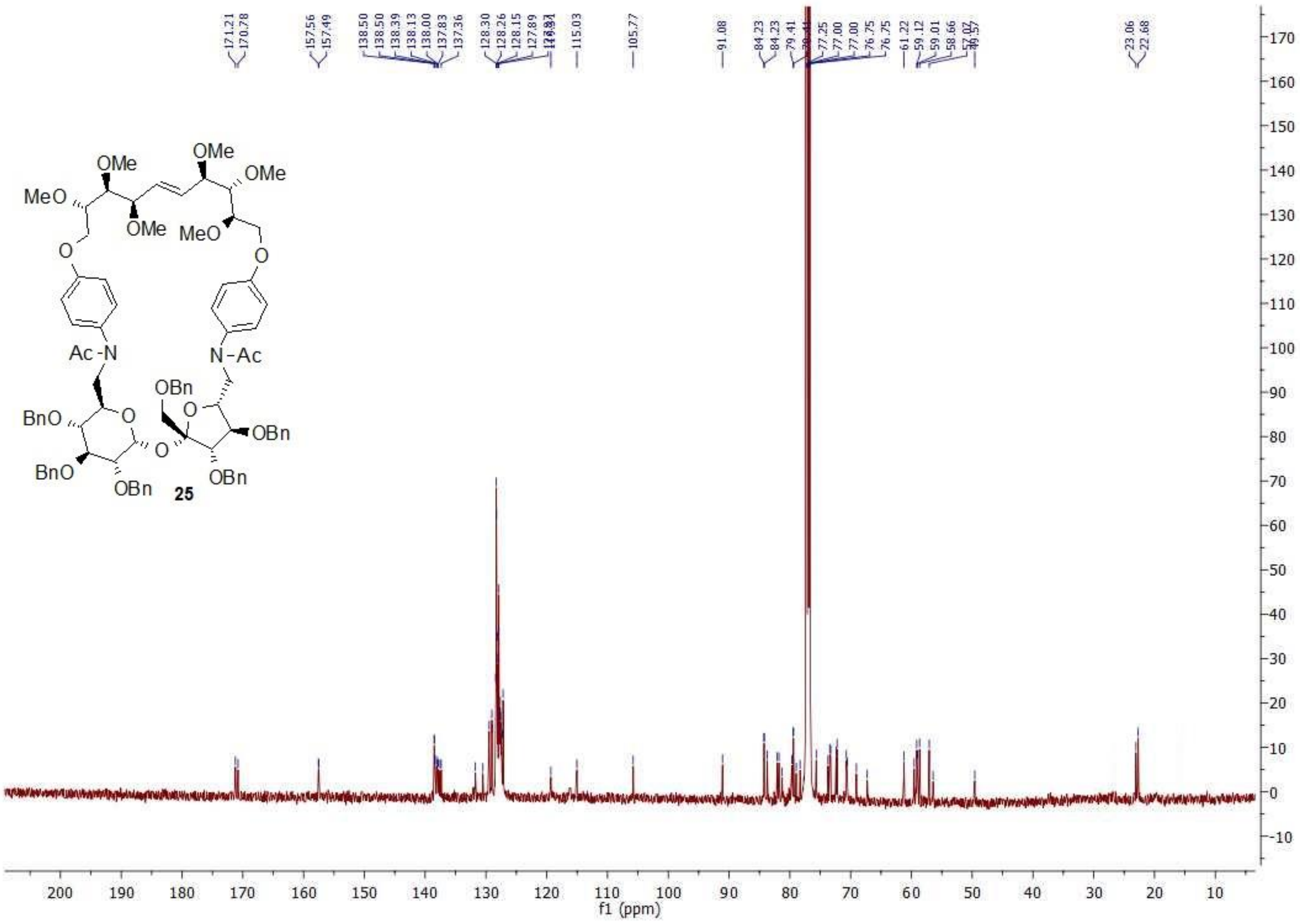

Figure S28 ^{13}C NMR spectrum of compound **25**.

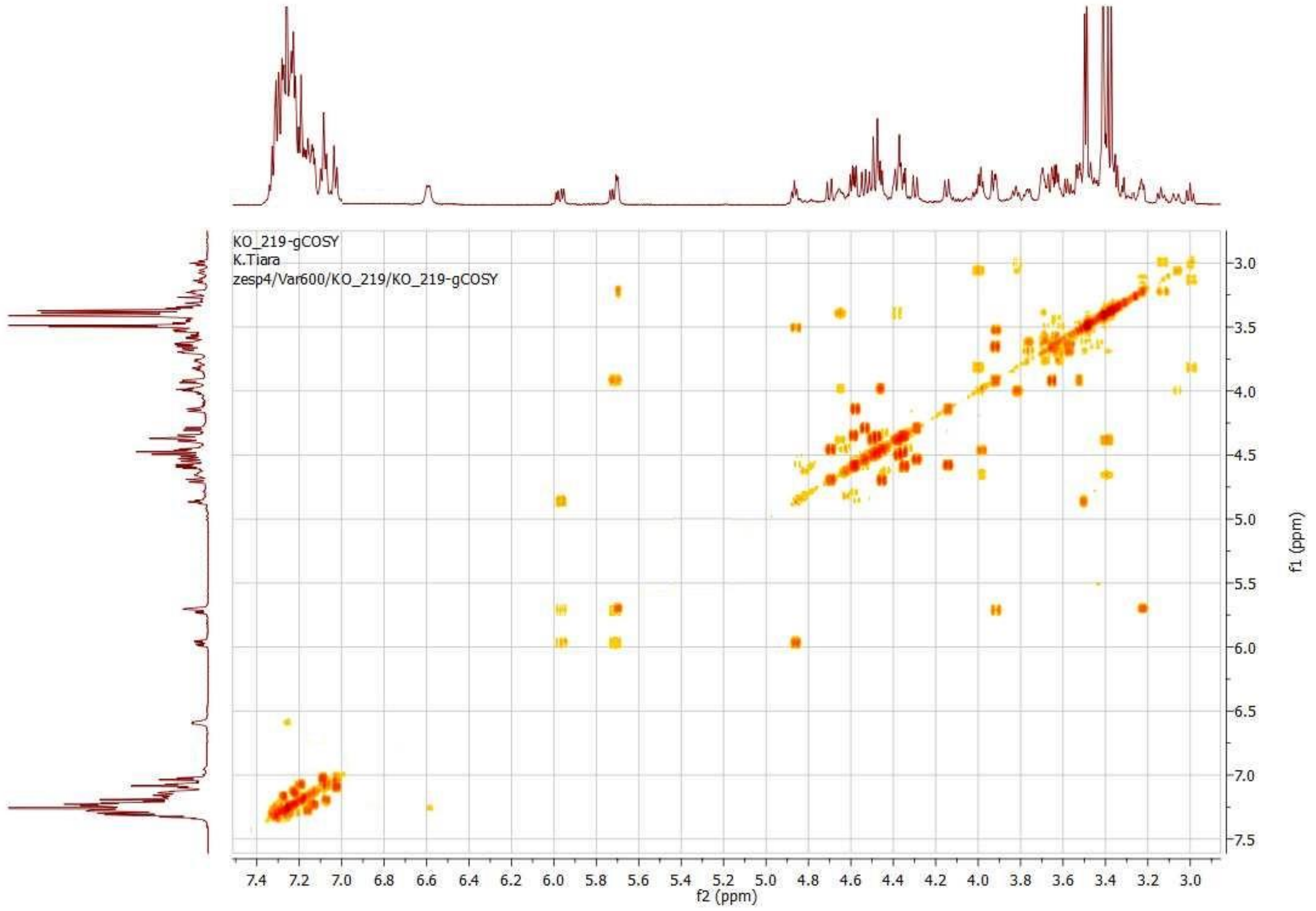

Figure S29 gCOSY spectrum of compound **25**.

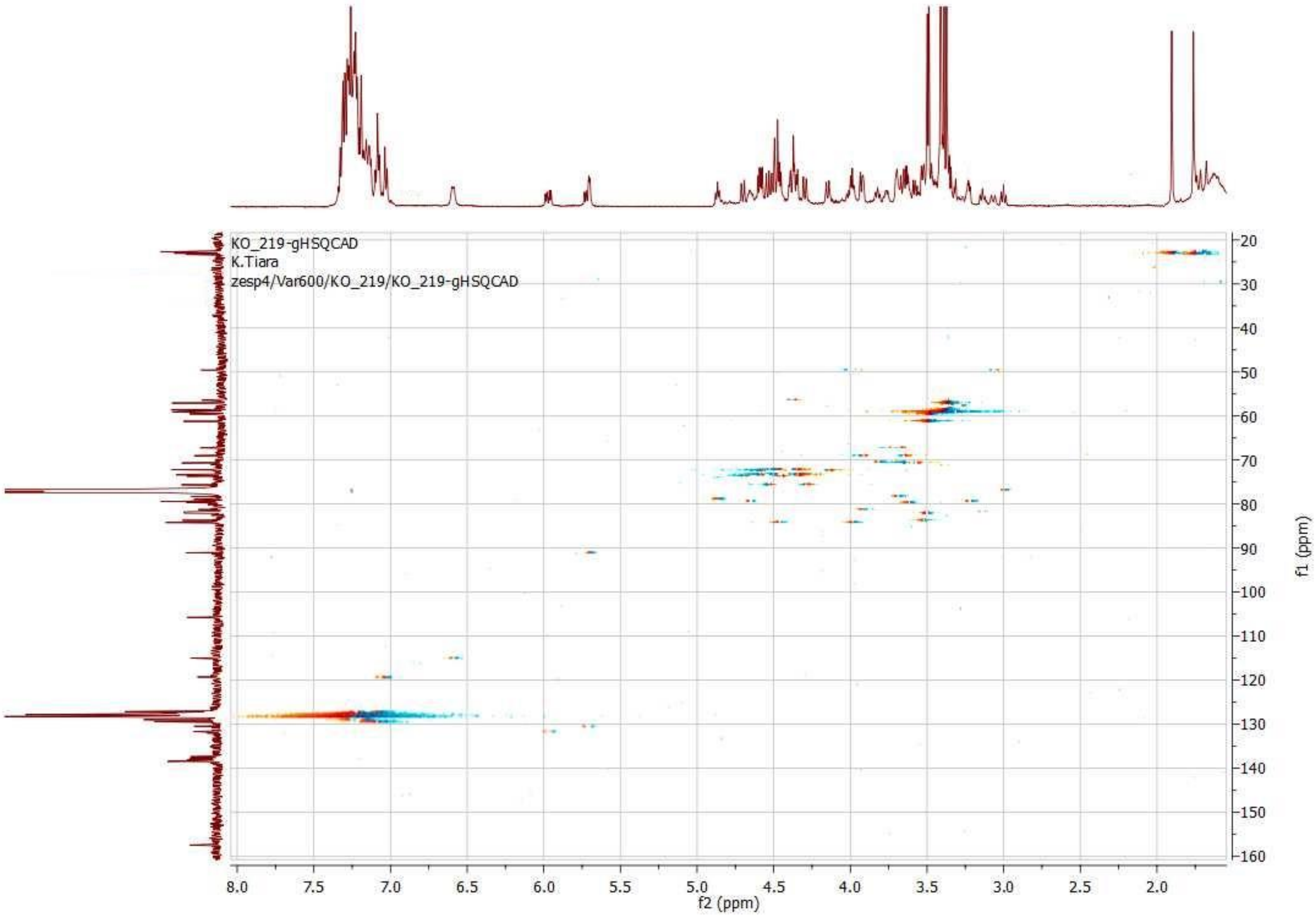

Figure S30 gHSQCAD spectrum of compound **25**.

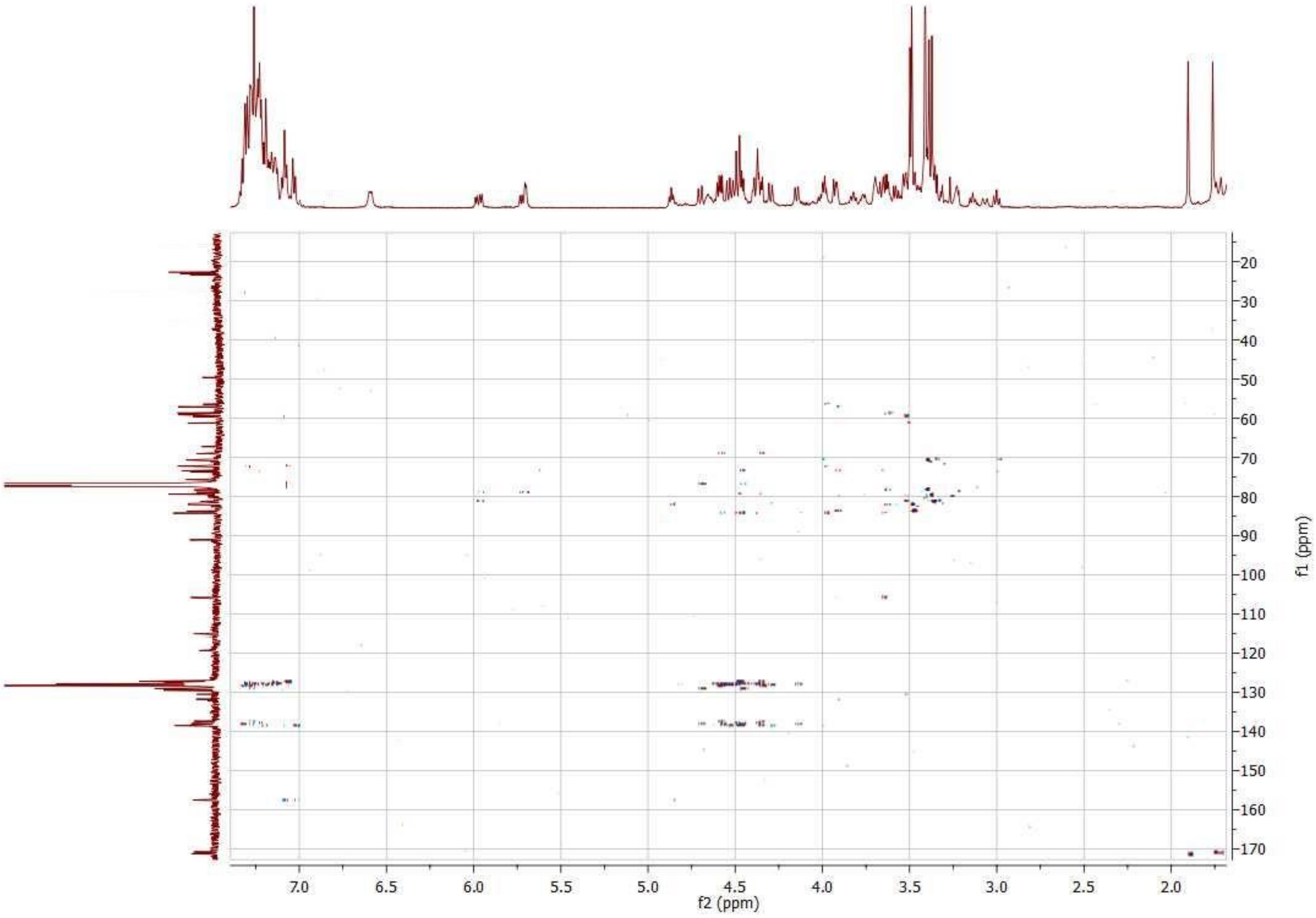

Figure S31 gHMBCAD NMR spectrum of compound **25**.
